# Harmonizing Perspectives on MPS II Care in Türkiye: A Delphi Study Towards Treatment Management Consensus

**DOI:** 10.3390/healthcare14091214

**Published:** 2026-04-30

**Authors:** Neslihan Onenli Mungan, Leyla Tumer, Serap Sivri, Nur Arslan, Sema Kalkan Ucar, Berna Seker Yilmaz, Gulden Gokcay

**Affiliations:** 1 Department of Pediatrics, Division of Pediatric Metabolism, Cukurova University Faculty of Medicine, Adana 01790, Türkiye; 2 Department of Pediatrics, Division of Pediatric Metabolism, Gazi University Faculty of Medicine, Ankara 06500, Türkiye; leylat@gazi.edu.tr; 3 Department of Pediatrics, Division of Pediatric Metabolism, Hacettepe University Faculty of Medicine, Ankara 06230, Türkiye; ssivri@hacettepe.edu.tr; 4 Department of Pediatrics, Division of Pediatric Metabolism, Dokuz Eylul University Faculty of Medicine, İzmir 35340, Türkiye; nur.arslan@ibg.edu.tr; 5 Department of Pediatrics, Division of Pediatric Metabolism, Ege University Faculty of Medicine, İzmir 35100, Türkiye; semakalkan@hotmail.com; 6 Genetics and Genomic Medicine, UCL Great Ormond Street Institute of Child Health, University College London, London WC1N 1EH, UK; b.yilmaz@ucl.ac.uk; 7 Department of Paediatric Inherited Metabolic Disease, Evelina Children’s Hospital, Guy’s and St Thomas’ NHS Foundation Trust, London SE1 7EH, UK; 8 Department of Pediatrics, Division of Pediatric Nutrition and Metabolism, Istanbul University Faculty of Medicine, Istanbul 34093, Türkiye; guldengokcay@gmail.com

**Keywords:** mucopolysaccharidosis type II (MPS II), Hunter syndrome, Delphi method, enzyme replacement therapy, rare disease consensus

## Abstract

**Background**: Mucopolysaccharidosis type II (MPS II; Hunter syndrome) is a rare X-linked lysosomal storage disorder caused by pathogenic variants in the *iduronate-2-sulfatase* gene, leading to progressive multisystem involvement. Although international management guidelines exist, challenges in their implementation across different healthcare systems remain insufficiently addressed. This study aimed to establish a national expert consensus in Türkiye on the treatment and management of MPS II, aligning local practice with international standards. **Methods**: A modified Delphi methodology was conducted using two rounds of online surveys supported by three steering committee meetings. The process involved 10 experienced clinicians and a scientific committee of six professors. Based on international guidelines and country-specific clinical challenges, 72 consensus statements and 84 exploratory questions were developed. Statements achieving ≥ 80% agreement were accepted as consensus. **Results**: Consensus supported initiating enzyme replacement therapy (ERT) in both severe and attenuated MPS II, guided by functional and cognitive status. Severe cognitive impairment was not considered an exclusion criterion for ERT, given its somatic benefits. Experts agreed on continuing ERT into adulthood with individualized discontinuation decisions. Routine evaluations every 6–12 months, including respiratory, cardiac, and neurocognitive assessments, were recommended. Additional consensus areas included individualized premedication strategies, structured transition to adult care, selective home infusion, annual patient-reported outcome assessments, and the establishment of a national MPS II registry. Hematopoietic stem cell transplantation was not endorsed. **Conclusions**: This Delphi study demonstrates strong expert consensus on MPS II management in Türkiye, providing a practical framework to guide clinical practice, support alignment with international recommendations, and inform future policy and research priorities.

## 1. Introduction

Mucopolysaccharidosis (MPS) type II, or Hunter Syndrome, is an X-linked recessive lysosomal storage disorder (LSD) caused by pathogenic variants in the *iduronate-2-sulfatase (IDS)* gene. This leads to reduced activity of the enzyme iduronate-2-sulfatase (I2S; OMIM 309900) and subsequent accumulation of the glycosaminoglycans (GAGs) dermatan sulfate and heparan sulfate, resulting in a wide range of clinical manifestations [1]. MPS II is the most common mucopolysaccharidosis in Europe, accounting for 29% of all MPS [2]. The estimated incidence ranges from 1 in 60,000 to 1 in 150,000 [3]. It primarily affects males, though rare female cases exist due to skewed X-chromosome inactivation [4,5].

MPS II exhibits substantial allelic and clinical heterogeneity, with classification schemes, including severe or attenuated, neuronopathic or non-neuronopathic, and early progressive or slowly progressive forms [1,6,7]. Approximately 60% of patients present with the severe, neuronopathic form—typically between ages 2 and 4—exhibiting behavioral issues, intellectual decline, and neurodegeneration. These patients often die in adolescence from airway obstruction or cardiac failure. In contrast, individuals with the attenuated form may retain normal or mildly impaired cognition and survive into late adulthood. Phenotypic consistency is common among siblings, and the clinical spectrum ranges from severe neurodegeneration to attenuated forms without neuronal involvement [8,9]. Diagnosis is often delayed due to nonspecific symptoms that overlap with common childhood conditions [10]. Clinical suspicion should be confirmed through biochemical and genetic tests, including urinary GAG excretion, I2S activity analysis, and identification of pathogenic *IDS* variants [10,11,12].

Enzyme replacement therapy (ERT) remains the current standard of care for MPS II and has been shown to improve somatic manifestations, including organomegaly, endurance, and pulmonary function [13,14,15]. However, its inability to cross the blood–brain barrier limits its impact on neurocognitive outcomes [16]. Alternative therapeutic approaches, including modified enzyme delivery systems, gene therapy, and other investigational strategies, are currently under evaluation, although their role in routine clinical practice remains to be established [17,18,19,20,21]. Hematopoietic stem cell transplantation (HSCT) has also been explored, but its efficacy in MPS II remains uncertain and is not widely adopted [19]. Despite these advances, significant unmet needs persist, particularly in addressing neurological involvement and optimizing long-term disease management.

Though international guidelines for MPS II management exist, applying them consistently across healthcare systems is challenging [11,12,22,23,24,25]. As a rare disease, MPS II’s prevalence and management vary between countries, influenced by differences in healthcare infrastructure, cultural factors, and resource availability. Türkiye, with its unique demographic and clinical landscape, provides a compelling setting to investigate these variations [26]. Although precise epidemiological data for MPS II in Türkiye are limited, national expert estimates and registry-based observations suggest that approximately 150–200 patients are currently under follow-up across specialized metabolic centers. The majority of diagnosed patients are receiving enzyme replacement therapy (ERT) with idursulfase as the standard of care, in line with reimbursement policies and international recommendations. While the disease is recognized by local clinicians and the Ministry of Health, limited information exists on how closely Turkish medical practice aligns with international recommendations, or what specific factors contribute to potential variations. For example, access to diagnostics and treatments may differ across regions, which can significantly affect patient journey and outcomes [26].

To optimize care delivery for MPS II in Türkiye, a better understanding of real-world practices and challenges is needed. While international consensus documents provide a foundation, they may not fully reflect local realities—such as resource constraints, variable physician experience with rare diseases, and individual patient needs. Capturing the perspectives of healthcare professionals experienced in managing MPS II is essential to fill these knowledge gaps. By distilling expert opinions, healthcare stakeholders can work toward more standardized treatment protocols that resonate with the Turkish healthcare reality, without contradicting existing evidence. To address these objectives, a Delphi method study has been initiated with the following aims: (1) to gather expert opinions on MPS II management in Türkiye, (2) to identify common and differentiating aspects of patient journey, and (3) to establish a consensus on best practices and recommendations tailored to the Turkish healthcare setting.

## 2. Materials and Methods

### 2.1. Study Design

We used a modified Delphi methodology to conduct this study. The Delphi method is a structured, systematic, and iterative approach designed to achieve expert consensus, especially when real-world evidence is scarce or inconclusive [27,28]. While there is no universally established standard regarding the optimal number of Delphi panelists or rounds, classical Delphi studies often comprise three or more rounds, whereas modified approaches, such as the one employed in this study, are sufficiently completed within two rounds of online surveys, fed by 2 steering committee meetings to assess the questions and answers and a final steering committee meeting [29]. In the present study, a high level of agreement was reached across most statements after the second round, and only minimal changes were observed between rounds, suggesting that additional rounds would be unlikely to significantly alter the outcomes.

The initial questionnaire was developed based on a literature review, real-world challenges, and unmet needs identified by a scientific steering committee of national experts. Statements were assessed using Likert scales with pre-defined consensus thresholds set at 80%, and questions were designed with multiple-choice formats.

### 2.2. Identification of Participants

A steering committee was established, composed of six professor-level academician specialists with extensive expertise in MPS II management, all of whom actively contribute to national or international metabolic or rare disease societies and guideline development. This committee was responsible for overseeing the scientific and methodological integrity of the study, including the finalization of the questionnaire and governance of the consensus process. Subsequently, a panel of 10 physicians from Türkiye was recruited. Selection criteria included having at least 10 years of experience managing patients with lysosomal storage disorders, authorship in relevant publications, and active involvement in clinical practice in university or research and training hospitals. To ensure diversity of expertise and minimize selection bias, panelists were recruited from multiple tertiary academic centers across different regions of Türkiye, all of which are actively involved in the diagnosis and management of lysosomal storage disorders. The selection process aimed to include clinicians with varied clinical experience, institutional backgrounds, and exposure to different patient populations, thereby enhancing the representativeness of real-world practice within the national context. Participation was voluntary, and all participants consented to the process.

### 2.3. Formulation of Questions and Assessment of Answers

Before the first Delphi round, the steering committee and an independent consultant conducted a focused literature review covering MPS II diagnosis, disease burden, treatment landscape, treatment optimization, and unmet medical needs. International guidelines and consensus documents were also systematically reviewed to ensure alignment with best practices. Based on this review, 72 statement questions were drafted, covering treatment decision-making, patient management pathways, enzyme replacement therapy (ERT) optimization, and supportive care considerations. In addition to academic statement questions, there were also 84 exploratory questions in the first online round, which aimed to evaluate real clinical practices and identify patterns that align with or diverge from consensus academic views. These questions were reviewed and approved by the steering committee to ensure clarity and clinical relevance.

**Figure 1:**
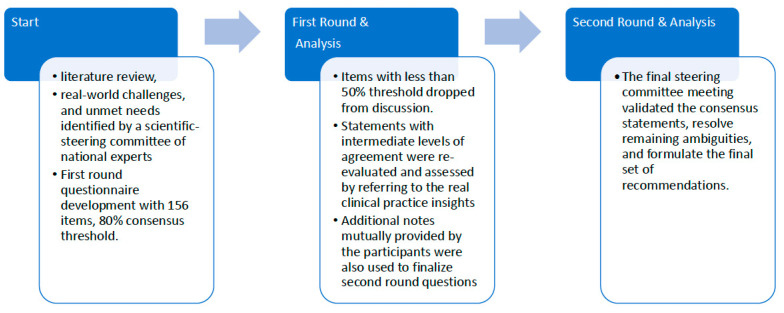
Delphi process.

Panelists responded to the statement questions in electronic questionnaires using Likert scales (1 = strongly disagree to 5 = strongly agree) and exploratory questions with multiple-choice formats with an open-ended choice to get their detailed insights/opinions if they had any. Statements achieving ≥ 80% agreement were considered to have reached consensus. Agreement was defined as responses of “agree” or “strongly agree” (scores of 4 or 5 on the Likert scale), while disagreement was defined as responses of “disagree” or “strongly disagree” (scores of 1 or 2). Items with less than 50% agreement were excluded from the second round. Statements with intermediate levels of agreement were re-evaluated and assessed by referring to the real clinical practice insights, then refined for the second online survey round based on panel feedback. Additional notes mutually provided by the participants were also used to finalize the second round questions. The final steering committee meeting served to validate the consensus statements, resolve remaining ambiguities, and formulate the final set of recommendations (Figure 1).

## 3. Results

### 3.1. Overall Results from First Round and Divergences in Clinical Practice

All participants responded to all questions in both online rounds (Files S1 and S2). After the first round, the steering committee analyzed all results, confirmed, and agreed on the consensus achieved statements, reassessed, and developed a report on near-consensus items as well as debatable statements due to divergent opinions and important insights reflecting significant variation from the provided academic opinions versus actual clinical practices (File S3). This report guided the development of new guidance statements for the second survey round, aiming to deepen consensus and facilitate the standardization and optimization of treatment management across Turkish clinical settings. The achieved consensus on all statements is given in the following sections, while we summarize the key clinical practice insights.

The outcome of the first-round survey confirmed strong consensus on key aspects like early ERT initiation, palliative care integration, and multidisciplinary care. Disagreements highlighted differing opinions on the necessity of ERT for mild or severe symptoms and the perceived role of palliative care. Variance in clinical practice also highlighted cautious administration of ERT for patients with severe neurological symptoms and cognitive impairment, while emerging therapies like gene therapy and substrate reduction therapy represented potential areas for a second round survey exploration to bridge gaps between academic insights and clinical practice. First round survey results also highlighted that measuring urinary GAG levels, liver and spleen ultrasounds, joint range of motion, and polysomnography assessments exhibit variability and ongoing divergence in practices. Respondents frequently aligned with their academic beliefs on semi-annual evaluations for follow-ups, spirometry, and 6-Minute Walk Tests. Otolaryngologic and ophthalmological follow-ups also align closely with academic expectations. Discrepancies in the frequency of urinary GAG measurements, liver and spleen size monitoring, polysomnography, and quality of life assessments highlighted areas where clinical practice diverges from academic recommendations. Topics such as ERT discontinuation for severe infusion-associated reactions, life-threatening comorbidities, and neurological decline showed variability among participants. Clear alignment was seen in areas like using overall health assessments for continuing ERT and recognizing that ERT is well-tolerated with mild to moderate side effects. Significant variations were observed in clinical decisions on ERT discontinuation due to life-threatening comorbidities, advanced disease progression, and neurological decline. Finally, it was observed that most of the participants do not discontinue or suspend ERT treatment in their clinical practice based on single assessment criteria but on overall clinical assessment/outcome. However, there is a significant divide in opinion on forced vital capacity (FVC), liver and spleen size/assessment frequency, and neurological decline, as half of the participants selected some criteria and timelines that the rest did not (Files S1 and S3).

### 3.2. Treatment Initiation

The Delphi panel reached substantial agreement on key aspects of treatment initiation for patients with MPS II in Türkiye. As summarized in Table 1, panelists endorsed clear criteria for when to initiate enzyme replacement therapy (ERT), considering clinical diagnosis, symptomatology, and disease progression. The consensus statements reflected a shared understanding of the importance of early intervention and harmonization with international guidelines, while also addressing country-specific challenges and practices. Consensus rates ranged from moderate to very high across the proposed statements, highlighting both areas of alignment and topics requiring further consideration in Turkish clinical practice.

### 3.3. Follow-Up Requirements

The Delphi panel reached substantial agreement on key elements of follow-up protocols for patients with MPS II (Table 2). Consensus was achieved on the multidisciplinary nature of follow-up, recommended assessment intervals, and core components of monitoring, including clinical, biochemical, and functional parameters. However, consensus rates varied more than in other domains, reflecting the recognized heterogeneity in follow-up practices both internationally and within the Turkish healthcare system.

### 3.4. Treatment Continuation

Experts agreed on key determinants for maintaining enzyme replacement therapy (ERT), including the evaluation of clinical benefit, progression of organ involvement, and the presence of safety concerns. The panel also aligned on situations where continuation of treatment is still recommended despite the presence of disease progression, provided that the benefit-risk ratio remains favorable (Table 3).

### 3.5. Treatment and System Optimization

Panelists agreed on several key aspects, including the need for individualized treatment planning, multidisciplinary care involvement, regular reassessment of disease status, and incorporation of patient-centered considerations into treatment decisions. The recommendations emphasize the importance of tailoring management strategies to the evolving needs of each patient, ensuring alignment with both international best practices and local clinical realities (Table 4).

## 4. Discussion

This study aimed to establish expert consensus on key aspects of MPS II management, including treatment initiation, follow-up, treatment continuation, and strategies for optimizing clinical care. It is important to emphasize that the findings of this Delphi study reflect real-world clinical practice and expert consensus within the Turkish healthcare setting. As such, the recommendations are shaped by local healthcare infrastructure, access to diagnostics and treatments, and accumulated clinical experience. In routine practice, variability in access to specialized diagnostic tools, follow-up infrastructure, and institutional practices may influence how international recommendations are interpreted and applied. While several consensus statements align with well-established international guidelines, their inclusion serves to confirm the consistency and applicability of these core principles within routine care. More importantly, the added value of this Delphi process lies in identifying areas where clinical practice is more variable or where evidence remains limited—particularly in follow-up strategies, monitoring approaches (e.g., urinary GAG assessment, imaging intervals), and complex treatment decisions such as continuation or discontinuation of therapy. These domains often rely on clinical judgment, making them well suited to expert consensus methodologies. Therefore, rather than aiming to generate universally generalizable recommendations or formally compare healthcare systems, this study provides practice-oriented guidance tailored to Türkiye, while also offering insights relevant to other settings facing similar real-world implementation challenges.

A strong consensus was reached regarding the initiation of enzyme replacement therapy (ERT) in patients with both severe and attenuated forms of MPS II, consistent with existing evidence and international recommendations [12,22,23,24,25]. The panel highlighted that ERT initiation should be guided by clinical factors such as mobility, respiratory function, and cognitive impairment, echoing the principles outlined in the MPS II guidelines. Notably, the panel endorsed the initiation of ERT in attenuated patients without neurodevelopmental deficits/before severe neurological symptoms appear, aligning with findings suggesting that early ERT can delay disease progression and improve functional outcomes even in patients with milder phenotypes.

The rejection of severe cognitive impairment (defined by low developmental quotient [DQ] scores) as an exclusion criterion for ERT (80% disagreement) is particularly noteworthy. While earlier studies have debated the limited efficacy of ERT in preventing neurocognitive decline due to poor blood-brain barrier penetration, emerging real-world data suggest that ERT may still confer somatic benefits and improve quality of life even in cognitively impaired patients [15,30,31]. Furthermore, the panel raised concerns regarding the validity of DQ assessments in MPS II, as comorbidities such as hearing loss, motor impairments, or behavioral issues may lead to falsely low scores. This is in line with recent discussions advocating for the cautious interpretation of cognitive testing in rare disease populations [32].

The consensus supporting the continuation of ERT into adolescence and adulthood reflects current clinical practice trends and the growing body of evidence suggesting that prolonged ERT contributes to the stabilization of somatic symptoms and sustained improvements in endurance and respiratory function [24,33,34]. The recommendation for treatment to be managed by metabolic specialists with specific expertise is also in harmony with international standards.

Interestingly, the panel expressed low agreement with the routine use of hematopoietic stem cell transplantation (HSCT) in attenuated MPS II (80% disagreement). Although HSCT has demonstrated some benefits in other lysosomal storage disorders such as MPS I, its role in MPS II remains controversial due to limited efficacy and increased procedural risks, including infection, transplant rejection, and graft-versus-host disease [19,35]. This cautious position reflects both the available evidence and the limited adoption of HSCT in MPS II clinical practice globally. The consensus also acknowledged the growing potential of emerging therapies, including gene therapy, substrate reduction therapy (SRT), and molecular Trojan horse strategies. The recognition of these modalities as promising or future options is well supported by ongoing clinical trials and early-phase studies reporting encouraging safety and efficacy data [36,37]. Nevertheless, their incorporation into routine care remains premature pending further validation.

The unanimous endorsement of a multidisciplinary, palliative, and psychosocial care approach underscores the panel’s recognition of the complex needs of patients and families affected by MPS II. This aligns with best practices recommended by international consensus documents emphasizing that optimal MPS II management extends beyond ERT to address emotional, educational, and supportive care needs [22,23,38].

The panel confirmed the importance of regular clinical evaluations, including medical history and physical examinations every 6 months with laboratory monitoring of glycosaminoglycan (GAG) levels at baseline and every 6 months, especially during the early phases of enzyme replacement therapy (ERT). This is consistent with international guidelines recommending frequent assessments during the first year of ERT to evaluate treatment responsiveness [11,12,22,25]. Additionally, consensus was reached on performing echocardiography, ECG, and abdominal ultrasound at baseline and annually, reflecting the high burden of cardiovascular, hepatic, and splenic involvement in MPS II. These recommendations are further supported by recent real-world data showing the impact of ERT on stabilizing organomegaly and cardiac abnormalities over long-term follow-up [15,24,39,40,41].

The panel strongly supported periodic functional assessments, such as the 6-Minute Walk Test (6MWT) and spirometry, which are essential for capturing changes in endurance, pulmonary function, and overall disease progression. The emphasis on cognitive assessments at regular intervals tailored to age and disease severity is particularly relevant given the neurocognitive heterogeneity of MPS II [6]. While cognitive decline is more common in the severe phenotype, even attenuated patients may develop subtle neurocognitive impairments over time [6,40,42]. Notably, the consensus to individualize cognitive follow-up frequency according to phenotypic severity and educational context is in line with recommendations from the American College of Medical Genetics and Genomics (ACMG) and other expert panels [22,23,25].

Joint range of motion and MRI assessments, particularly focusing on cervical spine pathology, were recognized as key components of follow-up [43,44,45]. This reflects the high prevalence of skeletal and spinal complications that can contribute to morbidity and reduced quality of life [46,47]. However, the recommendation to perform cervical spine MRI every 24 months in patients with MPS II is still debated internationally, as some authors suggest that annual imaging may be preferable for patients with rapidly progressing disease [11,22,25].

Consensus was achieved on the need for regular ear, nose, throat (ENT), and ophthalmologic evaluations, which are essential to detect common complications such as hearing loss, airway obstruction, and vision impairment. These issues are often underdiagnosed but significantly affect the quality of life in MPS II patients [22,48,49,50].

The relatively lower consensus (70%) regarding routine IgG monitoring reflects ongoing debates about its role in the long-term management of MPS II. While infusion-related reactions and immunogenicity to ERT (idursulfase) have been reported in several studies, the clinical significance of antibody formation, particularly the impact of anti-idursulfase IgG on treatment efficacy, remains uncertain [51]. Some studies suggest that high-titer antibodies may contribute to reduced clinical benefit or infusion reactions, while others have not confirmed a strong correlation [51,52,53]. Therefore, although some experts recommend baseline and periodic IgG monitoring, especially in cases of poor clinical response or allergic reactions, its routine use is still not universally adopted. Furthermore, in some countries, such as Türkiye, the measurement of anti-idursulfase IgG is not routinely available, which may also contribute to the lack of widespread implementation of regular monitoring practices. This likely explains the moderate consensus observed among the panel.

Additionally, the unanimous agreement on the annual use of PRO questionnaires underscores the increasing recognition of the importance of capturing patient and caregiver perspectives, which complements objective clinical assessments and has been advocated in recent literature [54,55]. However, consensus rates varied more than in other domains, reflecting the recognized heterogeneity in follow-up practices both internationally and within the Turkish healthcare system, particularly due to limited access to imaging facilities for annual echocardiograms and MRIs, and inconsistent adherence to recommended urinary GAG testing schedules. This variability underscores the challenges of harmonizing follow-up recommendations in rare diseases, where evidence is often limited, and expert opinion becomes a critical driver of clinical guidance [56]. These results offer a valuable foundation for adapting follow-up strategies to the Turkish context while aligning with global principles of care.

The panel unanimously agreed that ERT is generally considered a life-long therapy for patients with MPS II, consistent with international recommendations and real-world evidence. This is consistent with studies demonstrating that long-term ERT contributes to the stabilization or improvement of somatic manifestations, particularly in respiratory, cardiac, hepatosplenic, and musculoskeletal systems [15,57]. The majority also agreed (80%) that the overall benefits of ERT outweigh its costs under most circumstances. However, the slightly lower agreement may reflect regional variations in healthcare resource allocation and access to therapy.

A strong consensus was reached regarding the rare but important scenarios in which ERT may be discontinued. The panel agreed that severe infusion-related reactions, unresponsiveness to optimal premedication and desensitization strategies, may justify ERT withdrawal. Similarly, discontinuation may be considered in cases of severe, life-threatening comorbidities where continuing ERT could worsen the patient’s condition or when the burden of therapy exceeds its expected benefits, especially in end-of-life settings [11,58].

The panel emphasized that inadequate response in isolated domains—such as endurance (6MWT), echocardiographic parameters, respiratory function, or organ size reduction—should not be interpreted as treatment failure nor serve as an automatic indication for discontinuation. This view is consistent with evidence showing that treatment benefits may vary across organ systems and may not always translate into measurable improvements in every functional parameter [59]. In addition, this recommendation helps prevent premature cessation of ERT based on isolated outcome measures and encourages a holistic assessment of the patient’s overall health.

The panel strongly endorsed that ERT continuation decisions should be individualized, considering the patient’s quality of life, personal and family preferences, and multidisciplinary team input. This patient-centered approach is aligned with best practice recommendations in rare disease management [60]. Given the absence of an alternative disease-modifying therapy for MPS II, the consensus reinforced that ERT remains the mainstay of treatment aimed at preserving or improving the patient’s quality of life over time.

The panel strongly agreed on the importance of individualizing premedication strategies and ensuring systematic management of hypersensitivity reactions associated with ERT. Although routine premedication is not mandatory for all patients, it is considered good clinical practice to tailor prophylaxis (e.g., antihistamines and corticosteroids) according to patient-specific infusion histories and risk factors. This reflects current international guidelines and real-world data, which suggest that tailored premedication can reduce infusion-related adverse events without unnecessarily complicating the treatment regimen [61,62]. Furthermore, the panel emphasized that hypersensitivity reactions and infusion-related adverse events should be thoroughly documented and standardized across centers, in alignment with previous expert recommendations.

Unanimous agreement was achieved regarding the potential for home infusion of ERT in carefully selected patients. This aligns with evidence supporting the safety, feasibility, and potential quality-of-life benefits of home-based ERT, provided that patients are clinically stable and emergency protocols are available. Home infusion programs have been successfully implemented in multiple countries, leading to improved patient satisfaction without compromising safety [63].

The panel highlighted the importance of a structured and well-coordinated transition process from pediatric to adult care services. Transition is a recognized challenge in lysosomal storage disorders (LSDs) due to the multisystemic nature of the disease and the shift from pediatric to adult-focused healthcare systems [64,65]. The consensus stressed that transition planning should be personalized, multidisciplinary, and supported by comprehensive documentation, ensuring continuity of care and safeguarding patient safety, especially in emergency situations. Despite broad agreement, a slightly lower consensus (80%) on certain transition items may reflect local healthcare system limitations or variability in transition protocols between institutions.

The panel achieved high consensus on the necessity of establishing a centralized and well-structured national registry for MPS II, including both clinical and genetic data. The registry was seen as critical for improving long-term understanding of disease progression, optimizing care delivery, and facilitating real-time clinical decision-making. Similar registries, such as the Hunter Outcome Survey (HOS) and other LSD registries, have demonstrated the value of high-quality longitudinal data in informing clinical practice, policymaking, and research [66,67]. Moreover, the consensus underscored the need for systematic audits, governance, and collaboration with international registries to ensure data accuracy, comparability, and long-term sustainability.

## 5. Conclusions

The findings confirm alignment with international guidelines on core principles such as early initiation of enzyme replacement therapy (ERT), multidisciplinary care, and individualized treatment decision-making based on clinical status and disease progression. Importantly, the study also highlights areas where clinical practice is more variable or where evidence remains limited—particularly in follow-up strategies and monitoring approaches, including the frequency and prioritization of urinary glycosaminoglycan (GAG) assessments, imaging intervals (e.g., MRI and echocardiography), and the use of functional evaluations such as the 6-Minute Walk Test (6MWT) and spirometry. Variability was also observed in complex clinical decision-making, especially regarding treatment continuation or discontinuation in the context of disease progression, comorbidities, or perceived treatment response. These domains reflect real-world clinical challenges where standardized evidence is lacking, and expert judgment plays a critical role in guiding patient management.

## 6. Strengths and Limitations of the Study

This study’s main strength lies in its ability to gather expert consensus from a group of Turkish professionals with extensive experience in managing MPS II, ensuring that the results are grounded in the clinical realities of treating this rare disease in Türkiye. The use of the Delphi methodology enabled a systematic approach to reaching consensus, allowing for expert opinions to be refined through multiple rounds of feedback. This process contributed to a reliable set of recommendations that reflect the current clinical practices for MPS II management, offering valuable insights for healthcare providers in the region. The study also provides comprehensive coverage of essential topics, such as treatment initiation, monitoring protocols, and treatment discontinuation, and highlights the importance of a national registry for improving patient care and outcomes. Furthermore, the inclusion of emerging therapies such as substrate reduction therapy, molecular Trojan horse strategies, and gene therapy reflects the ongoing evolution of treatment strategies in the field.

Despite its strengths, this study has several limitations that should be considered when interpreting the findings. First, the relatively small number of Delphi panelists (n = 10), although consistent with methodological standards for rare disease expert panels, may limit the generalizability of the findings. However, all participants were highly experienced clinicians from leading centers managing MPS II in Türkiye, which supports the validity and relevance of the consensus generated. Second, the selection of panelists based on expertise and academic involvement may introduce potential selection bias, as participants may share similar clinical perspectives or practice environments. To mitigate this, efforts were made to include experts from different institutions and regions, ensuring representation of diverse clinical experiences within the Turkish healthcare system. Third, as the study was funded by industry, there is a theoretical risk of sponsor-related bias. However, multiple safeguards were implemented to minimize this risk. The scientific steering committee maintained full independence in study design, questionnaire development, data interpretation, and formulation of conclusions. The sponsor had no role in the conduct of the Delphi rounds, analysis of responses, or decision to publish, ensuring the scientific integrity of the process. Additionally, the study did not incorporate patient or caregiver perspectives, which represent an important dimension in rare disease management. While the Delphi methodology focused on clinical expert consensus, the absence of patient-reported experiences may limit the comprehensiveness of the recommendations, particularly in areas related to quality of life and treatment burden. Future studies integrating patient and caregiver input would provide a more holistic understanding of MPS II management. Lastly, while the study discusses emerging therapies, these options remain largely theoretical, and the lack of long-term clinical data means that the recommendations should be reconsidered as further research becomes available.

## 7. Future Directions and Implications for Research and/or Practice

Through the Delphi process, the study aims to establish a unified front among Turkish clinicians, setting the stage for ongoing collaboration and research. By reaching a consensus on essential items for optimizing treatment—whether those entail adjusting diagnostic protocols, enhancing patient monitoring strategies, or advocating for wider access to ERT—the findings will help shape an actionable framework for MPS II management in Türkiye. The hope is that such a framework will strengthen patient care pathways, improve the quality of life for individuals affected by MPS II, and serve as a model for other regions addressing similar challenges in the management of rare diseases.

## Figures and Tables

**Table 1:** Consensus Statements on Treatment Initiation.

Statements on Treatment Initiation	Consensus Rate *
ERT should be started on patients who have attenuated form without neurodevelopmental deficits or disorders.	80% agreement
ERT should be started on patients who have severe form of the disease with mild neurological symptoms.	100% agreement
The initiation of ERT in MPS II should be based on certain criteria such as mobility, respiratory functions, and cognitive impairment.	80% agreement
ERT should be started in MPS II patients over 24 months of age who have mobility without assistance and do not require respiratory support in the last six months, as part of the criteria for treatment eligibility.	80% agreement
Eligibility for ERT should be decided on a case-by-case basis	80% agreement
Severe cognitive decline (developmental quotient [DQ] < 70) (DQ < 70) should be an exclusion criterion for ERT in patients over 2 years of age.	80% disagreement
DQ is not a suitable criterion for assessing cognitive impairment in children with MPS II, considering they may have various disabilities that can mislead DQ scores, and even healthy children may score lower depending on the testing conditions.	90% agreement
Treatment should be carried out under the supervision of metabolic specialists with experience and expertise in the treatment of MPS II and ERT.	100% agreement
ERT is the current standard of care.	90% agreement
ERT is indicated for the long-term treatment of patients with MPS II, during puberty and after reaching adulthood.	100% agreement
Hematopoietic Stem Cell Transplantation (HSCT) should be considered a viable treatment option for patients with attenuated MPS II.	80% disagreement
HSCT should be considered a viable treatment option in patients with severe MPS II.	80% agreement
Substrate reduction therapy is a promising option for MPS II based on small-molecule inhibitors of GAG synthesis, which prevents substrate storage.	80% agreement
Gene therapy is an emerging treatment for MPS II and may replace ERT in the future.	100% agreement
Palliative care should be integrated into the care plan of MPS II patients from the time of diagnosis to address physical, emotional, and psychological needs.	100% agreement
Emotional and psychological support for both the patient and their family should be a core component of palliative care in MPS II.	100% agreement
Multidisciplinary team-based care should be the standard approach for managing MPS II.	100% agreement

* Consensus was defined as when 80% to 100% of the panel members marked the “agree/strongly agree” or “disagree/strongly disagree” option.

**Table 2:** Consensus Statements on Follow-up Requirements.

Statements	Consensus Rate *
The medical history evaluations and physical examinations should be performed every 6 months (except case-specific circumstances).	90% agreement
It is good clinical practice to monitor urinary GAG levels at baseline and repeat every 6 months for the first year of ERT to track initial response and extend to 12 months once the patient’s clinical and biochemical markers have stabilized, if there is adequate clinical and test access capacity.	100% agreement
It is a good clinical practice to measure liver and spleen size by ultrasound at baseline and repeat every 12 months if there is adequate clinical/imaging access capacity (except case-specific circumstances where a more often monitoring is required).	100% agreement
It is good clinical practice to perform the 6-Minute Walk Test (6MWT) at baseline and repeat every 12 months.	90% agreement
It is good clinical practice to perform spirometry at baseline and repeat every 12 months if there is adequate clinical/test access capacity.	90% agreement
It is good clinical practice to perform cognitive assessments in patients with MPS II every 6–12 months until 3 years of age depending on phenotype severity and any emerging concerns.	100% agreement
It is good clinical practice to perform cognitive assessments in patients with MPS II every 12 months between 3–5 years of age if stable, more often if there are signs of rapid changes.	100% agreement
It is good clinical practice to perform cognitive assessments in patients with MPS II every 12–18 months between 5–12 years of age, focusing on academic performance, attention, adaptive functioning, and social development.	100% agreement
It is good clinical practice to perform cognitive assessments in patients with MPS II every 2 years from 13 years of age, individualized to the patient’s clinical course and school/vocational needs.	100% agreement
It is good clinical practice to perform echocardiograms and ECGs at baseline and repeat every 12 months if there is adequate clinical/test access capacity (except case-specific circumstances where more often monitoring is required).	100% agreement
Joint range of motion should be assessed at baseline and repeated every 12 months if there is adequate clinical capacity (except case-specific circumstances where more often monitoring is required).	90% agreement
It is good clinical practice to perform MRI of the spine (particularly cervical) and the brain at baseline and repeat every 24 months for patients with MPS II if there is adequate imaging capacity (except case-specific circumstances where more often monitoring is required.)	100% agreement
It is good clinical practice to perform otolaryngologic follow-up at baseline and repeat every 12 months if there is adequate clinical capacity (except case-specific circumstances where more often monitoring is required).	100% agreement
It is good clinical practice to perform ophthalmological examination with fundus assessments at baseline and repeat every 12 months if there is adequate clinical capacity (except case-specific circumstances).	100% agreement
It is good clinical practice to perform IgG monitoring at baseline and repeat annually or more often if clinical response is inadequate or due to allergic reactions if there is adequate clinical/test access capacity.	70% ** agreement
It is good clinical practice to perform polysomnography at baseline and repeat annually in case of sleep apnea or nocturnal snoring.	90% agreement
Quality of life questionnaires should be administered annually to evaluate the impact of ERT on patients’ overall well-being.	100% agreement

* Consensus was defined as when 80% to 100% of the panel members marked the “agree/strongly agree” or “disagree/strongly disagree” option. ** Close to consensus item.

**Table 3:** Consensus Statements on Treatment Continuation.

*Statements on Treatment Continuation*	Consensus Rate *
The apparent benefits of ERT outweigh the costs under any circumstance.	80% agreement
ERT is a life-long therapy for MPS II.	90% agreement
ERT should be discontinued or suspended when there is a severe infusion-associated reaction that cannot be managed with recommended premedication and desensitization.	100% agreement
ERT should be discontinued or suspended when there are secondary life-threatening comorbidities (review on a case-by-case basis).	90% agreement
While there is not enough evidence to directly recommend if ERT must be discontinued or suspended during pregnancy or lactation in MPS II, if the clinical benefit is significant, continuing therapy—with appropriate monitoring—can be reasonable. Ultimately, the decision is individualized.	90% agreement
Deciding whether to continue or suspend ERT in a patient with an unrelated, terminal illness depends on the overall goals of care, quality of life considerations, and the projected benefits of therapy. Ultimately, the decision is individualized, balancing the risks and benefits in consultation with the specialist team.	90% agreement
Inadequate response in 6MWT follow up after any length of ERT should not solely be considered as treatment failure	100% agreement
Inadequate response in Echocardiographic functions follow up after any length of ERT should not solely be considered as treatment failure nor indicate treatment discontinuation.	100% agreement
Inadequate response in FVC follow up after any length of ERT should not solely be considered as a treatment failure nor indicate treatment discontinuation.	100% agreement
Inadequate response on liver or spleen size or volume up after any length of ERT should not solely be considered as treatment failure nor indicate treatment discontinuation.	100% agreement
Individualized and overall assessment, together with the physician’s, patient’s and the family’s opinion should be the determinants of ERT discontinuation.	100% agreement
ERT continuation is important due to the absence of any other specific treatment. The criteria should be regulated in a positive way to improve the patient’s quality of life.	100% agreement

* Consensus was defined as when 80% to 100% of the panel members marked the “agree/strongly agree” or “disagree/strongly disagree” option.

**Table 4:** Consensus Statements on Clinical Management Optimization.

Statements	Consensus Rate *
While premedication and desensitization to prevent hypersensitivity reactions in ERT is not mandatory for all cases, it is good clinical practice to individualize protocols, tailor to the patient’s infusion-related reaction history, comorbidities, and risk factors.	100% agreement
Antihistamines and corticosteroids are essential components of the premedication regimen before ERT in MPS II patients to prevent hypersensitivity reactions.	90% agreement
Side effects related to ERT in MPS II, such as hypersensitivity reactions, should be carefully documented and reviewed to optimize patient care.	100% agreement
Immediate intervention strategies, such as slowing the infusion rate or administering rescue medications, should be standardized in the management of ERT-related side effects.	100% agreement
Post-infusion monitoring should be mandatory for a specific period after ERT to detect and manage any delayed side effects.	90% agreement
Patients should be educated on recognizing potential ERT side effects and when to seek immediate medical attention.	100% agreement
Home infusion of ERT for MPS II can be considered for children older than 2 years of age, provided they have had no significant infusion reactions in the preceding 6 months and there is a trained healthcare professional available to manage potential emergencies.	100% agreement
Patients should be transitioned to adult clinics with a well-organized transfer process.	80% agreement
Mutual visits should be organized to enable both the adult care and pediatric care physicians to share the disease history and the specificity of the patient’s condition.	80% agreement
Accurate preparation of medical documentation is crucial during the adult transition process. Properly organized medical documentation ensures smooth information flow and continuity of care as patients transition to adult healthcare services. Therefore, this documentation should contain key information on the patient’s previous and current health condition, therapeutic recommendations, and algorithms for management in the case of a life-threatening event.	100% agreement
Establishing a registry is crucial for obtaining long-term results and understanding the impact on health economics.	90% agreement
A centralized registry system for MPS II should be implemented to avoid duplication of patient data across different regions or healthcare facilities.	90% agreement
The MPS II registry should include both clinical and genetic data to ensure a comprehensive understanding of the disease and its progression.	90% agreement
The MPS II registry should allow for real-time data entry and access by healthcare professionals to facilitate timely decision-making in patient management.	90% agreement
Regular audits and reviews of the MPS II registry should be conducted to ensure data quality, accuracy, and relevance to ongoing patient care and research.	100% agreement
The national medical association should lead the initiative in overseeing and maintaining the MPS II registry and monitor the disease together with the health authority to ensure data accuracy, compliance with national standards with ethical measures and permissions taken.	100% agreement
Involvement of patient associations is crucial for raising awareness, encouraging patient enrollment, and improving the effectiveness of the MPS II registry.	80% agreement
International collaboration should be encouraged to ensure that the MPS II registry aligns with global standards and facilitates cross-border research efforts.	100% agreement
The success of the MPS II registry depends on the provision of adequate funding and resources to support long-term maintenance and expansion.	90% agreement

* Consensus was defined as when 80% to 100% of the panel members marked the “agree/strongly agree” or “disagree/strongly disagree” option.

## Data Availability

This study is based on expert consensus data collected through online surveys (via SurveyMonkey) and a face-to-face meeting. All relevant data generated and analyzed during the Delphi process are included in the Supplementary Materials (File S1: MPS-2 Delphi First Round; File S2: MPS-2 Delphi Second Round; File S3: MPS-2 Delphi First Round Report). Further inquiries can be directed to the corresponding author.

## Supplementary Materials

The following supporting information can be downloaded at: https://www.mdpi.com/article/10.3390/healthcare14091214/s1. File S1: MPS-2 Delphi First Round; File S2: MPS-2 Delphi Second Round; File S3: MPS-2 Delphi First Round Report.

## Abbreviations

The following abbreviations are used in this manuscript:

**Abbreviation**	**Definition**
ACMG	American College of Medical Genetics and Genomics
CNS	Central Nervous System
DQ	Developmental Quotient
ECG	Electrocardiogram
ENT	Ear, Nose, and Throat
ERT	Enzyme Replacement Therapy
FDA	Food and Drug Administration
GAG	Glycosaminoglycan
GT	Gene Therapy
HSCT	Hematopoietic Stem Cell Transplantation
*IDS*	Iduronate-2-sulfatase gene
I2S	Iduronate-2-sulfatase enzyme
LV	Lentiviral Vector
LSD	Lysosomal Storage Disorder
MPS II	Mucopolysaccharidosis Type II
MRI	Magnetic Resonance Imaging
OMIM	Online Mendelian Inheritance in Man
PRO	Patient-Reported Outcome
SRT	Substrate Reduction Therapy
Türkiye	Türkiye (Republic of Turkey)
6MWT	6-Minute Walk Test
