# Supplementary material for: Harmonizing Perspectives on MPS II Care in Türkiye: A Delphi Study Towards Treatment Management Consensus
Source: Healthcare (Basel). 2026 Apr 30;14(9):1214. doi: 10.3390/healthcare14091214 (PMC13164175; doi:10.3390/healthcare14091214)
Supplement: Supplementary file 1 [file healthcare-14-01214-s001.zip › File S1_MPS-2_Delphi_first round.pdf]

Dear Participating Physician,

The aim of this first set of questions in MPS-2 is to understand your current treatment management views/opinions as well as your own treatment management approach for treating MPS-2 patients in your clinical practice. In each section **first your general opinions/believes are asked** to look for a consensus among the statement answers by Delphi Methodology, then additional **current clinical practice** questions are also asked to understand your current clinical practices in your clinic and how they may vary from your academic views and clinical guidelines as each clinical setting/country may have variations in terms of access to clinics, assessments as well as the criteria for treatment eligibility.

Please don't hesitate to contact Remedium team members if you need any additional clarification to answer this questionnaire.

Thank you once again for your time and invaluable inputs for this project.

Best Regards

PART-1

CONSIDERATIONS ON MPS-2 TREATMENT INITIATION: WHEN AND WHOM TO TREAT?

1. ERT should be introduced as early as possible after diagnosis.

☐I strongly agree ☐I agree ☐ Undecided / Neutral ☐I disagree ☐I strongly disagree

2. Early initiation of enzyme replacement therapy (ERT) is of key importance as it offers a chance to slow down the disease progression and can prevent the irreversible effects of the disease.

☐I strongly agree ☐I agree ☐ Undecided / Neutral ☐I disagree ☐I strongly disagree

3. ERT should only be started patients older than five years of age.

☐I strongly agree ☐I agree ☐ Undecided / Neutral ☐I disagree ☐I strongly disagree

4. ERT should be started to all children and adolescents regardless of age.

☐I strongly agree ☐I agree ☐ Undecided / Neutral ☐I disagree ☐I strongly disagree

5. ERT should be started to patients who have attenuated form without neurodevelopmental deficits or disorders.

☐I strongly agree ☐I agree ☐ Undecided / Neutral ☐I disagree ☐I strongly disagree

6. ERT should be started to patients who have severe form of the disease with neurological symptoms.

☐I strongly agree ☐I agree ☐ Undecided / Neutral ☐I disagree ☐I strongly disagree

7. ERT should be started to all patients regardless of the severity and CNS involvement.

☐I strongly agree ☐I agree ☐ Undecided / Neutral ☐I disagree ☐I strongly disagree

8. Eligibility for ERT should be decided on a case-by-case basis.

☐I strongly agree ☐I agree ☐ Undecided / Neutral ☐I disagree ☐I strongly disagree

9. Severe cognitive decline (DQ<70) should be an exclusion criterion for ERT in patients over 2 years of age.

☐I strongly agree ☐I agree ☐ Undecided / Neutral ☐I disagree ☐I strongly disagree

10. DQ is not a suitable criterion for assessing cognitive impairment in children with MPS II, considering they may have various disabilities that can mislead DQ scores, and even normal children may score lower depending on the testing conditions.

☐I strongly agree ☐I agree ☐ Undecided / Neutral ☐I disagree ☐I strongly disagree

11. ERT should be started regardless of any criteria at any age.

☐I strongly agree ☐I agree ☐ Undecided / Neutral ☐I disagree ☐I strongly disagree

12. The initiation of ERT in MPS II should be based on criteria including mobility, respiratory functions and/or cognitive impairment.

☐I strongly agree ☐I agree ☐ Undecided / Neutral ☐I disagree ☐I strongly disagree

13. Please feel free to provide additional comments/share your opinions on the above clinical practice insight statement interms of access to experts/ expert clinics/clinical infrastructure in your country or simply please choose the "No additional comment" option.
    a. --------------------------------------
    b. No additional comment

14. ERT should be started in MPS II patients over 24 months of age who has mobility without assistance and do not require respiratory support in the last six months, as part of the criteria for treatment eligibility.

☐I strongly agree ☐I agree ☐ Undecided / Neutral ☐I disagree ☐I strongly disagree

15. Treatment should be carried out under the supervision of metabolic specialists with experience and expertise in the treatment of MPS II and ERT.

☐I strongly agree ☐I agree ☐ Undecided / Neutral ☐I disagree ☐I strongly disagree

16. ERT is the current standard of care.

☐ I strongly agree ☐ I agree ☐ Undecided / Neutral ☐ I disagree ☐ I strongly disagree

17. ERT is indicated for the long-term treatment of patients with MPS II, during puberty and after reaching adulthood.

☐ I strongly agree ☐ I agree ☐ Undecided / Neutral ☐ I disagree ☐ I strongly disagree

18. Haematopoietic Stem Cell Transplantation (HSCT) should be considered a viable treatment option for patients with attenuated MPS II.

☐I strongly agree ☐I agree ☐ Undecided / Neutral ☐I disagree ☐I strongly disagree

19. HSCT should be considered a viable treatment option in patients with severe MPS II.

☐I strongly agree ☐I agree ☐ Undecided / Neutral ☐I disagree ☐I strongly disagree

20. HSCT is unlikely to provide benefit to patients with MPS II.
☐I strongly agree ☐I agree ☐ Undecided / Neutral ☐I disagree ☐I strongly disagree

21. HSCT is likely to provide benefit to patients with MPS II.
☐I strongly agree ☐I agree ☐ Undecided / Neutral ☐I disagree ☐I strongly disagree

22. ERT should be continued in MPS II patients prior to undergoing HSCT to maintain enzyme levels.

☐I strongly agree ☐I agree ☐ Undecided / Neutral ☐I disagree ☐I strongly disagree

23. ERT should be continued for at least 6 months after HSCT in MPS II patients to ensure stability while monitoring the transplant outcome.

☐I strongly agree ☐I agree ☐ Undecided / Neutral ☐I disagree ☐I strongly disagree

24. Substrate reduction therapy is a promising option for MPSII based on small-molecule inhibitors of GAG synthesis, which prevents substrate storage.
☐I strongly agree ☐I agree ☐ Undecided / Neutral ☐I disagree ☐I strongly disagree

25. Gene therapy is an emerging treatment for MPSII and may replace ERT in the future.
☐I strongly agree ☐I agree ☐ Undecided / Neutral ☐I disagree ☐I strongly disagree

26. Palliative care should be integrated into the care plan of MPS II patients from the time of diagnosis to address physical, emotional, and psychological needs.
☐ I strongly agree ☐ I agree ☐ Undecided / Neutral ☐ I disagree ☐ I strongly disagree

27. Emotional and psychological support for both the patient and their family should be a core component of palliative care in MPS II.

☐ I strongly agree ☐ I agree ☐ Undecided / Neutral ☐ I disagree ☐ I strongly disagree

28. Multidisciplinary team-based care should be the standard approach for managing MPS II.

☐ I strongly agree ☐ I agree ☐ Undecided / Neutral ☐ I disagree ☐ I strongly disagree

Individual practices/opinions/insights

29. In my clinic, I start ERT as early as possible after diagnosis.
☐Always ☐Often ☐Sometimes ☐Rarely ☐Never

30. Please feel free to provide additional comments/share your opinions on the above clinical practice insight statement interms of access to experts/ expert clinics/clinical infrastructure in your country or simply please choose the “No additional comment” option.
    a. --------------------------------------
    b. No additional comment
31. In my clinic, I start ERT only for patients older than five years of age.
☐Always ☐Often ☐Sometimes ☐Rarely ☐Never
32. Please feel free to provide additional comments/share your opinions on the above clinical practice insight statement interms of access to experts/ expert clinics/clinical infrastructure in your country or simply please choose the “No additional comment” option.
    c. --------------------------------------
    d. No additional comment

33. In my clinic, I start ERT to all children and adolescents regardless of age.
☐Always ☐Often ☐Sometimes ☐Rarely ☐Never
34. Please feel free to provide additional comments/share your opinions on the above clinical practice insight statement interms of access to experts/ expert clinics/clinical infrastructure in your country or simply please choose the “No additional comment” option.
    a. --------------------------------------
    b. No additional comment
35. In my clinic, I start ERT to patients who have attenuated form without neurodevelopmental deficits or disorders.
☐Always ☐Often ☐Sometimes ☐Rarely ☐Never
36. Please feel free to provide additional comments/share your opinions on the above clinical practice insight statement in terms of access to experts/ expert clinics/clinical infrastructure in your country or simply please choose the “No additional comment” option.
    a. --------------------------------------
    b. No additional comment

37. In my clinic, I start ERT to patients who have severe form of the disease with neurological symptoms.
☐Always ☐Often ☐Sometimes ☐Rarely ☐Never

38. Please feel free to provide additional comments/share your opinions on the above clinical practice insight statement in terms of access to experts/ expert clinics/clinical infrastructure in your country or simply please choose the “No additional comment” option.
    a. --------------------------------------
    b. No additional comment

39. In my clinic, I start ERT to patients regardless of the severity and CNS involvement.
☐Always ☐Often ☐Sometimes ☐Rarely ☐Never

40. Please feel free to provide additional comments/share your opinions on the above clinical practice insight statement in terms of access to experts/ expert clinics/clinical infrastructure in your country or simply please choose the “No additional comment” option.
    a. --------------------------------------
    b. No additional comment

41. In my clinic, I decide on the eligibility for ERT on a case-by-case basis.
☐Always ☐Often ☐Sometimes ☐Rarely ☐Never

42. Please feel free to provide additional comments/share your opinions on the above clinical practice insight statement in terms of access to experts/ expert clinics/clinical infrastructure in your country or simply please choose the “No additional comment” option.
    a. --------------------------------------
    b. No additional comment

43. In my clinic practice, cognitive decline (DQ<70) is an exclusion criterion for ERT in patients over 2 years of age.
☐Always ☐Often ☐Sometimes ☐Rarely ☐Never

44. Please feel free to provide additional comments/share your opinions on the above clinical practice insight statement in terms of access to experts/ expert clinics/clinical infrastructure in your country or simply please choose the “No additional comment” option.

a. --------------------------------------

b. No additional comment

45. In my clinic, I start ERT regardless of cognitive impairment at any age.
▢Always ▢Often ▢Sometimes ▢Rarely ▢Never

46. Please feel free to provide additional comments/share your opinions on the above clinical practice insight statement in terms of access to experts/ expert clinics/clinical infrastructure in your country or simply please choose the "No additional comment" option.

    a. --------------------------------------

    b. No additional comment

47. In my clinic, ERT is the current standard of care.
▢Always ▢Often ▢Sometimes ▢Rarely ▢Never

48. Please feel free to provide additional comments/share your opinions on the above clinical practice insight statement or please choose the "No additional comment" option.

a. --------------------------------------

b. No additional comment

49. In my clinic, I administer ERT via weekly intravenous infusion at a dose of 0.5 mg/kg body weight.
▢Always ▢Often ▢Sometimes ▢Rarely ▢Never

50. Please feel free to provide additional comments/share your opinions on the above clinical practice insight statement or please choose the "No additional comment" option.

    a. --------------------------------------

    b. No additional comment

51. In my clinic, I use ERT for the long-term treatment of patients with MPS II, during puberty and after reaching adulthood.
▢Always ▢Often ▢Sometimes ▢Rarely ▢Never

52. Please feel free to provide additional comments/share your opinions on the above clinical practice insight statement or please choose the “No additional comment” option.

    a. --------------------------------------

    b. No additional comment

53. I consider HSCT as a viable treatment option for patients with attenuated MPS II.

☐Always ☐Often ☐Sometimes ☐Rarely ☐Never

54. Please feel free to provide additional comments/share your opinions on the above clinical practice insight statement interms of access to experts/ expert clinics/clinical infrastructure in your country or simply please choose the “No additional comment” option..

    a. --------------------------------------

    b. No additional comment

55. I consider HSCT as a viable treatment option in patients with severe MPS II.

☐Always ☐Often ☐Sometimes ☐Rarely ☐Never

56. Please feel free to provide additional comments/share your opinions on the above clinical practice insight statement in terms of access to experts/ expert clinics/clinical infrastructure in your country or simply please choose the “No additional comment” option.

    a. --------------------------------------

    b. No additional comment

57. I use substrate reduction therapy as a promising option for MPSII based on small-molecule inhibitors of GAG synthesis, which prevents substrate storage.

☐Always ☐Often ☐Sometimes ☐Rarely ☐Never

58. Please feel free to provide additional comments/share your opinions on the above clinical practice insight statement in terms of access to experts/ expert clinics/clinical infrastructure in your country or simply please choose the “No additional comment” option.

    a. --------------------------------------

b. No additional comment

59. I would use gene therapy for my MPSII patients and replace the ongoing enzyme replacement therapies if it was available.

☐Always ☐Often ☐Sometimes ☐Rarely ☐Never

60. I consider integrating palliative care into the care plan of MPS II patients from the time of diagnosis essential to address physical, emotional, and psychological needs.

☐ Always ☐ Often ☐ Sometimes ☐ Rarely ☐ Never

61. I believe that emotional and psychological support for both the patient and their family should be a core component of palliative care in MPS II.

☐ Always ☐ Often ☐ Sometimes ☐ Rarely ☐ Never

62. I consider multidisciplinary team-based care the standard approach for managing MPS II.

☐ Always ☐ Often ☐ Sometimes ☐ Rarely ☐ Never

63. Please feel free to provide additional comments/share your opinions on the above clinical practice insight statement in terms of access to experts/ expert clinics/clinical infrastructure in your country or simply please choose the "No additional comment" option.

    a. ---------------------------------------
    b. No additional comment

PART-2

CONSIDERATIONS ON FOLLOW UP AND MONITORING.

64. How often should outcomes of ERT be evaluated?
☐ Every 6 months ☐ Every 12 months ☐ Every 18 months ☐ Every 24 months ☐ Other: ___________

65. How often should medical history evaluations and physical examinations be performed?
☐ Every 6 months ☐ Every 12 months ☐ Every 18 months ☐ Every 24 months ☐ Other: ___________

66. How often should urinary GAG levels be measured?
☐ Every 6 months ☐ Every 12 months ☐ Every 18 months ☐ Every 24 months ☐ Other: ___________

67. How often should liver and spleen size be measured by ultrasound?
☐ Every 6 months ☐ Every 12 months ☐ Every 18 months ☐ Every 24 months ☐ Other: ___________

68. How often should the 6-Minute Walk Test (6MWT) be performed?
☐ Every 6 months ☐ Every 12 months ☐ Every 18 months ☐ Every 24 months ☐ Other: ___________

69. How often should spirometry be performed?
☐ Every 6 months ☐ Every 12 months ☐ Every 18 months ☐ Every 24 months ☐ Other: ___________

70. How often should cognitive assessments be conducted?
☐ Every 6 months ☐ Every 12 months ☐ Every 18 months ☐ Every 24 months ☐ Other: ___________

71. How often should echocardiograms and ECGs be performed?
☐ Every 6 months ☐ Every 12 months ☐ Every 18 months ☐ Every 24 months ☐ Other: ___________

72. How often should joint range of motion be assessed?
☐ Every 6 months ☐ Every 12 months ☐ Every 18 months ☐ Every 24 months ☐ Other: ___________

73. How often should an annual MRI of the CNS be performed for patients with MPS II?
☐ Every 6 months ☐ Every 12 months ☐ Every 18 months ☐ Every 24 months ☐ Other: ___________

74. How often should an annual MRI of the spine be performed for patients with MPS II?

☐ Every 6 months ☐ Every 12 months ☐ Every 18 months ☐ Every 24 months ☐ Other:

75. How often should annual otolaryngologic follow-up be recommended?
☐ Every 6 months ☐ Every 12 months ☐ Every 18 months ☐ Every 24 months ☐ Other: ___________

76. How often should annual ophthalmological examinations with fundus assessments be conducted?
☐ Every 6 months ☐ Every 12 months ☐ Every 18 months ☐ Every 24 months ☐ Other: ___________

77. How often should regular monitoring of Immunoglobulin G (IgG) antibodies to ERT be done?
☐ Every 6 months ☐ Every 12 months ☐ Every 18 months ☐ Every 24 months ☐ Other: ___________

78. How often should annual polysomnography be performed to assess sleep quality and respiratory function in patients receiving ERT?
☐ Every 6 months ☐ Every 12 months ☐ Every 18 months ☐ Every 24 months ☐ Other: ___________

79. How often should quality of life questionnaires be administered to evaluate the impact of ERT on patients' overall well-being?
☐ Every 6 months ☐ Every 12 months ☐ Every 18 months ☐ Every 24 months ☐ Other: ___________

Individual practices/opinions/insights

80. In my clinic, to evaluate the outcomes of ERT, periodic follow-up visits are performed:
☐ Every 6 months ☐ Every 12 months ☐ Every 18 months ☐ Every 24 months ☐ Other: ___________

81. In my clinic, medical history evaluations and physical examinations are performed:
☐ Every 6 months ☐ Every 12 months ☐ Every 18 months ☐ Every 24 months ☐ Other: ___________

82. In my clinic, urinary GAG levels are measured:
☐ Every 6 months ☐ Every 12 months ☐ Every 18 months ☐ Every 24 months ☐ Other: ___________

83. In my clinic, liver and spleen size are measured by ultrasound:
☐ Every 6 months ☐ Every 12 months ☐ Every 18 months ☐ Every 24 months ☐ Other: ___________

84. In my clinic, liver and spleen size are measured by MRI:
☐ Every 6 months ☐ Every 12 months ☐ Every 18 months ☐ Every 24 months ☐ Other: ___________

85. In my clinic, the 6-Minute Walk Test (6MWT) is performed:
☐ Every 6 months ☐ Every 12 months ☐ Every 18 months ☐ Every 24 months ☐ Other: ___________

86. In my clinic, spirometry is performed:
☐ Every 6 months ☐ Every 12 months ☐ Every 18 months ☐ Every 24 months ☐ Other: ___________

87. In my clinic, cognitive assessments are conducted:
☐ Every 6 months ☐ Every 12 months ☐ Every 18 months ☐ Every 24 months ☐ Other: ___________

88. In my clinic, echocardiograms and ECGs are performed:
☐ Every 6 months ☐ Every 12 months ☐ Every 18 months ☐ Every 24 months ☐ Other: ___________

89. In my clinic, joint range of motion assessments are conducted:
☐ Every 6 months ☐ Every 12 months ☐ Every 18 months ☐ Every 24 months ☐ Other: ___________

90. In my clinic, an annual MRI of the CNS is performed for patients with MPS II:
☐ Every 6 months ☐ Every 12 months ☐ Every 18 months ☐ Every 24 months ☐ Other: ___________

91. In my clinic, an annual MRI of the spine is performed for patients with MPS II:
☐ Every 6 months ☐ Every 12 months ☐ Every 18 months ☐ Every 24 months ☐ Other: ___________

92. In my clinic, annual otolaryngologic follow-up is recommended:
☐ Every 6 months ☐ Every 12 months ☐ Every 18 months ☐ Every 24 months ☐ Other: ___________

93. In my clinic, annual ophthalmological examinations with fundus assessments are conducted:
☐ Every 6 months ☐ Every 12 months ☐ Every 18 months ☐ Every 24 months ☐ Other: ___________

94. In my clinic, Immunoglobulin G (IgG) antibodies to idursulfase are measured:
☐ Every 6 months ☐ Every 12 months ☐ Every 18 months ☐ Every 24 months ☐ Other: ___________

95. In my clinic, regular monitoring of Immunoglobulin G (IgG) antibodies to idursulfase is done:

☐ Every 6 months ☐ Every 12 months ☐ Every 18 months ☐ Every 24 months ☐ Other: ___________

96. In my clinic, annual polysomnography is performed to assess sleep quality and respiratory function in patients receiving ERT:

☐ Every 6 months ☐ Every 12 months ☐ Every 18 months ☐ Every 24 months ☐ Other: ___________

97. In my clinic, quality of life questionnaires are administered to evaluate the impact of ERT on patients' overall well-being:

☐ Every 6 months ☐ Every 12 months ☐ Every 18 months ☐ Every 24 months ☐ Other: ___________

98. Please feel free to provide additional comments/share your opinions on the above clinical practice insight statement interms of access to experts/ expert clinics/clinical infrastructure in your country or simply please choose the “No additional comment” option.
    a. ---------------------------------------
    b. No additional comment

PART-3

CONSIDERATIONS ON MPS-2 TREATMENT DISCONTINUATION

99. The apparent benefits of ERT outweigh the costs under any circumstance.
☐I strongly agree ☐I agree ☐ Undecided / Neutral ☐I disagree ☐I strongly disagree

100. ERT is a life-long therapy for MPSII.
☐I strongly agree ☐I agree ☐ Undecided / Neutral ☐I disagree ☐I strongly disagree

101. ERT should be discontinued or suspended when there is a severe infusion-associated reactions that cannot be managed with recommended premedication.
☐I strongly agree ☐I agree ☐ Undecided / Neutral ☐I disagree ☐I strongly disagree

102. ERT should be discontinued or suspended when there are life-threatening comorbidities (review on a case-by-case basis).
☐I strongly agree ☐I agree ☐ Undecided / Neutral ☐I disagree ☐I strongly disagree

103. ERT should be discontinued or suspended when there is pregnancy or breastfeeding.
☐I strongly agree ☐I agree ☐ Undecided / Neutral ☐I disagree ☐I strongly disagree

104. ERT should be discontinued or suspended when there is incurable disease unrelated to Hunter syndrome (e.g., terminal cancer)
☐I strongly agree ☐I agree ☐ Undecided / Neutral ☐I disagree ☐I strongly disagree

105. ERT should be discontinued or suspended when there is severe or advanced disease that does not improve with ERT.
☐I strongly agree ☐I agree ☐ Undecided / Neutral ☐I disagree ☐I strongly disagree

106. 6MWT should be a criterion for treatment discontinuation. Do you agree?
☐ I strongly agree ☐ I agree ☐ Undecided / Neutral ☐ I disagree ☐ I strongly disagree

107. What is the optimal length of treatment with ERT before an inadequate response in the 6MWT may be considered as treatment failure?

☐ 6 months ☐ 12 months ☐ 18 months ☐ 24 months ☐ Other: __________

108. There should be an ongoing improvement or stabilization in 6MWT to continue ERT.

☐I strongly agree ☐I agree ☐ Undecided / Neutral ☐I disagree ☐I strongly disagree

109. What decline in the annual 6MWT indicates that ERT should be discontinued?

☐ More than 30% ☐ More than 20% ☐ More than 10% ☐ Other: __________

110. Decision for continuing ERT should not be based on only 6MWT changes, an overall assessment including growth, organ involvement, quality of life should all be considered.

☐I strongly agree ☐I agree ☐ Undecided / Neutral ☐I disagree ☐I strongly disagree

111. ERT should be discontinued if neurological decline progressing to a severe degree.

☐I strongly agree ☐I agree ☐ Undecided / Neutral ☐I disagree ☐I strongly disagree

112. Echocardiographic functions (ejection fraction, fractional shortening, and myocardium thickness) should be criteria for treatment discontinuation. Do you agree?

☐ I strongly agree ☐ I agree ☐ Undecided / Neutral ☐ I disagree ☐ I strongly disagree

113. What is the optimal length of treatment with ERT before an inadequate response in echocardiographic functions (ejection fraction, fractional shortening, and myocardium thickness) may be considered as treatment failure?

☐ 6 months ☐ 12 months ☐ 18 months ☐ 24 months ☐ Other: __________

114. What decline in echocardiographic functions (ejection fraction, fractional shortening, or myocardium thickness) indicates that ERT should be discontinued?

☐ More than 30% ☐ More than 20% ☐ More than 10% ☐ Other: __________

115. Respiratory function tests (FVC) should be criteria for treatment discontinuation. Do you agree?

☐ I strongly agree ☐ I agree ☐ Undecided / Neutral ☐ I disagree ☐ I strongly disagree

116. What is the optimal length of treatment with ERT before an inadequate response in respiratory function tests (FVC) may be considered as treatment failure?

☐ 6 months ☐ 12 months ☐ 18 months ☐ 24 months ☐ Other: __________

117. What decline in FVC indicates that ERT should be discontinued?

☐ More than 30% ☐ More than 20% ☐ More than 10% ☐ Other: __________

118. Changes in liver and spleen size and volume should be criteria for treatment discontinuation. Do you agree?

☐ I strongly agree ☐ I agree ☐ Undecided / Neutral ☐ I disagree ☐ I strongly disagree

119. What is the optimal length of treatment with ERT before an inadequate response in liver and spleen size and volume may be considered as treatment failure?

☐ 6 months ☐ 12 months ☐ 18 months ☐ 24 months ☐ Other: __________

120. What increase in liver or spleen size or volume indicates that ERT should be discontinued?

☐ More than 30% ☐ More than 20% ☐ More than 10% ☐ Other: __________

121. An overall health assessment incorporating the aforementioned criteria should be utilized to determine the continuation of ERT.

☐ I strongly agree ☐ I agree ☐ Undecided / Neutral ☐ I disagree ☐ I strongly disagree

122. ERT continuation should be decided individually rather than based on standardized criteria.

☐ I strongly agree ☐ I agree ☐ Undecided / Neutral ☐ I disagree ☐ I strongly disagree

Individual practices/opinions/insights of responders

123. In my clinic, I discontinue or suspend ERT when there is a severe infusion-associated reactions that cannot be managed with recommended premedication.
☐Always ☐Often ☐Sometimes ☐Rarely ☐Never

124. Please feel free to provide additional comments/share your opinions on the above clinical practice insight statement in terms of your own experience **or** please choose the "No additional comment" option.

a. --------------------------------------
b. No additional comment

125. In my clinic, I discontinue or suspend ERT when there are life-threatening comorbidities (review on a case-by-case basis).

▢Always ▢Often ▢Sometimes ▢Rarely ▢Never

126. Please feel free to provide additional comments/share your opinions on the above clinical practice insight statement in terms of. your own experience **or** please choose the "No additional comment" option.

a. --------------------------------------

b. No additional comment

127. In my clinic, I discontinue or suspend ERT when there is pregnancy or breastfeeding.

▢Always ▢Often ▢Sometimes ▢Rarely ▢Never

128. Please feel free to provide additional comments/share your opinions on the above clinical practice insight statement in terms of. your own experience **or** please choose the "No additional comment" option.

a. --------------------------------------

b. No additional comment

129. In my clinic, I discontinue or suspend ERT when there is incurable disease unrelated to Hunter syndrome (e.g., terminal cancer)

▢Always ▢Often ▢Sometimes ▢Rarely ▢Never

130. Please feel free to provide additional comments/share your opinions on the above clinical practice insight statement in terms of. your own experience **or** please choose the "No additional comment" option.

a. --------------------------------------

b. No additional comment

131. In my clinic, I discontinue or suspend ERT when there is severe or advanced disease that does not improve with ERT.
▢Always ▢Often ▢Sometimes ▢Rarely ▢Never

132. Please feel free to provide additional comments/share your opinions on the above clinical practice insight statement in terms of. your own experience **or** please choose the “No additional comment” option.

a. --------------------------------------

b. No additional comment

133. In my clinic, I discontinue ERT if there is a decline in echocardiographic functions (ejection fraction, fractional shortening, and myocardium thickness) of more than:
☐ 30% ☐ 20% ☐ 10%

134. In my clinic, I discontinue ERT if no measurable effect is seen in echocardiographic functions after:
☐ 6 months ☐ 12 months ☐ 18 months ☐ 24 months ☐ Other: ___________

135. In my clinic, I discontinue ERT if there is a decline in 6MWT of more than:
☐ 30% ☐ 20% ☐ 10%

136. In my clinic, I discontinue ERT if no measurable effect is seen in 6MWT after:
☐ 6 months ☐ 12 months ☐ 18 months ☐ 24 months ☐ Other: ___________

137. In my clinic, I discontinue ERT if there is a decline in FVC of more than:
☐ 30% ☐ 20% ☐ 10%

138. In my clinic, I discontinue ERT if no measurable effect is seen in respiratory function tests (FVC) after:
☐ 6 months ☐ 12 months ☐ 18 months ☐ 24 months ☐ Other: ___________

139. In my clinic, I discontinue ERT if there is an increase inlLiver and spleen size and volume of more than:
☐ 30% ☐ 20% ☐ 10%

140. In my clinic, I discontinue ERT if no measurable effect is seen in liver and spleen size and volume after:
☐ 6 months ☐ 12 months ☐ 18 months ☐ 24 months ☐ Other: ___________

141. My decision for continuing ERT is be based on only 6MWT changes, an overall assessment including growth, organ involvement, quality of life should all be considered.

▢Always ▢Often ▢Sometimes ▢Rarely ▢Never

a. No additional comment

142. In my clinic, I discontinue ERT if neurological decline progressing to a severe degree.

▢Always ▢Often ▢Sometimes ▢Rarely ▢Never

143. I observe that ERT is well tolerated and most side effects are mild to moderate.

▢Always ▢Often ▢Sometimes ▢Rarely ▢Never

144. Please feel free to provide additional comments/share your opinions on the above clinical practice insight statement in terms of your own experience **or** please choose the "No additional comment" option.

a. ---------------------------------------
b. No additional comment

Part 4

PREMEDICATION FOR ERT IN MPS II:

145. Premedication should be routinely administered before ERT in MPS II patients to reduce the risk of infusion-related reactions.
☐ I strongly agree ☐ I agree ☐ Undecided / Neutral ☐ I disagree ☐ I strongly disagree

146. Antihistamines and corticosteroids are essential components of the premedication regimen before ERT in MPS II patients to prevent hypersensitivity reactions.
☐ I strongly agree ☐ I agree ☐ Undecided / Neutral ☐ I disagree ☐ I strongly disagree

147. Standardized protocols for premedication in ERT for MPS II should be established and followed across all treatment centers to ensure consistent patient care.
☐ I strongly agree ☐ I agree ☐ Undecided / Neutral ☐ I disagree ☐ I strongly disagree

148. Premedication protocols should be personalized for each patient, considering their medical history and previous reactions to ERT.
☐ I strongly agree ☐ I agree ☐ Undecided / Neutral ☐ I disagree ☐ I strongly disagree

149. Monitoring the efficacy of premedication protocols through regular patient feedback and clinical evaluation is critical to reducing adverse reactions to ERT.
☐ I strongly agree ☐ I agree ☐ Undecided / Neutral ☐ I disagree ☐ I strongly disagree

150. Please feel free to provide additional comments/share your opinions on the above premedication statement in terms of your own experience/best practice examples/future vision or choose the "No additional comment" option.
   a. ____
   b. No additional comment

SIDE EFFECT MANAGEMENT IN ERT FOR MPS II:

151. Side effects related to ERT in MPS II, such as hypersensitivity reactions, should be carefully documented and reviewed to optimize patient care.
☐ I strongly agree ☐ I agree ☐ Undecided / Neutral ☐ I disagree ☐ I strongly disagree

152. Immediate intervention strategies, such as slowing the infusion rate or administering rescue medications, should be standardized in the management of ERT-related side effects.
☐ I strongly agree ☐ I agree ☐ Undecided / Neutral ☐ I disagree ☐ I strongly disagree

153. Healthcare providers should receive regular training on the management of ERT-related side effects, including how to handle anaphylactic reactions.

☐ I strongly agree ☐ I agree ☐ Undecided / Neutral ☐ I disagree ☐ I strongly disagree

154. Post-infusion monitoring should be mandatory for a specific period after ERT to detect and manage any delayed side effects.

☐ I strongly agree ☐ I agree ☐ Undecided / Neutral ☐ I disagree ☐ I strongly disagree

155. Patients should be educated on recognizing potential ERT side effects and when to seek immediate medical attention.

☐ I strongly agree ☐ I agree ☐ Undecided / Neutral ☐ I disagree ☐ I strongly disagree

156. Please feel free to provide additional comments/share your opinions on the above side effect management statement in terms of your own experience/best practice examples/future vision or choose the "No additional comment" option.

a. ____

b. No additional comment

Part 5

CONSIDERATIONS ON IMPROVING CLINICAL MANAGEMENT APPROACH, ADHERENCE AND PERSISTENCE.

157. Home infusion of ERT should be considered for patients who have received several months of treatment in the clinic and who are well-tolerating infusions.
☐I strongly agree ☐I agree ☐ Undecided / Neutral ☐I disagree ☐I strongly disagree

158. Home infusion of ERT should be considered for children older than 2 years of age.
☐I strongly agree ☐I agree ☐ Undecided / Neutral ☐I disagree ☐I strongly disagree

159. Home infusion of ERT should be considered for children older than 5 years of age.
☐I strongly agree ☐I agree ☐ Undecided / Neutral ☐I disagree ☐I strongly disagree

160. Home infusion of ERT should not be an option for children.
☐I strongly agree ☐I agree ☐ Undecided / Neutral ☐I disagree ☐I strongly disagree

161. Patients should be transitioned to adult clinics with a well organized transfer process.
☐I strongly agree ☐I agree ☐ Undecided / Neutral ☐I disagree ☐I strongly disagree

162. Mutual visits should be organized to enable both the adult care and pediatric care physicians to share the disease history and the specificity of the patient's condition.
☐I strongly agree ☐I agree ☐ Undecided / Neutral ☐I disagree ☐I strongly disagree

163. Please feel free to provide additional comments/share your opinions on the above **home infusion insight** statement in terms of your own experience/best practice examples/future vision **or** please choose the "No additional comment" option.

a. ---------------------------------------

No additional comment

164. Accurate preparation of medical documentation is crucial during the adult transition process. Properly organized medical documentation ensures smooth information flow and continuity of care as patients transition to adult healthcare services. Therefore, this documentation should contain key information on the patient's previous and current health condition, therapeutic recommendations, and algorithms for management in the case of a life-threatening event.

☐I strongly agree ☐I agree ☐ Undecided / Neutral ☐I disagree ☐I strongly disagree

165. Establishing a registry is crucial for obtaining long-term results and understanding the impact on health economics.

☐I strongly agree ☐I agree ☐ Undecided / Neutral ☐I disagree ☐I strongly disagree

166. The MPS II registry should be established through collaboration between healthcare professionals, patient associations, and national health authorities.

☐ I strongly agree ☐ I agree ☐ Undecided / Neutral ☐ I disagree ☐ I strongly disagree

167. A centralized registry system for MPS II should be implemented to avoid duplication of patient data across different regions or healthcare facilities.

☐ I strongly agree ☐ I agree ☐ Undecided / Neutral ☐ I disagree ☐ I strongly disagree

168. The national medical association should lead the initiative in overseeing and maintaining the MPS II registry to ensure data accuracy and compliance with national standards.

☐ I strongly agree ☐ I agree ☐ Undecided / Neutral ☐ I disagree ☐ I strongly disagree

169. Involvement of patient associations is crucial for raising awareness, encouraging patient enrollment, and improving the effectiveness of the MPS II registry.

☐ I strongly agree ☐ I agree ☐ Undecided / Neutral ☐ I disagree ☐ I strongly disagree

170. The MPS II registry should include both clinical and genetic data to ensure a comprehensive understanding of the disease and its progression.

☐ I strongly agree ☐ I agree ☐ Undecided / Neutral ☐ I disagree ☐ I strongly disagree

171. The MPS II registry should allow for real-time data entry and access by healthcare professionals to facilitate timely decision-making in patient management.

☐ I strongly agree ☐ I agree ☐ Undecided / Neutral ☐ I disagree ☐ I strongly disagree

172. Regular audits and reviews of the MPS II registry should be conducted to ensure data quality, accuracy, and relevance to ongoing patient care and research.

☐ I strongly agree ☐ I agree ☐ Undecided / Neutral ☐ I disagree ☐ I strongly disagree

173. International collaboration should be encouraged to ensure that the MPS II registry aligns with global standards and facilitates cross-border research efforts.

☐ I strongly agree ☐ I agree ☐ Undecided / Neutral ☐ I disagree ☐ I strongly disagree

174. The success of the MPS II registry depends on the provision of adequate funding and resources to support long-term maintenance and expansion.

☐ I strongly agree ☐ I agree ☐ Undecided / Neutral ☐ I disagree ☐ I strongly disagree

175. Please feel free to provide additional comments/share your opinions on the above statement in terms of your own experience/best practice examples/future vision or choose the "No additional comment" option.

a. ____

b. No additional comment

END of FIRST ROUND SURVEY
