# Supplementary material for: Harmonizing Perspectives on MPS II Care in Türkiye: A Delphi Study Towards Treatment Management Consensus
Source: Healthcare (Basel). 2026 Apr 30;14(9):1214. doi: 10.3390/healthcare14091214 (PMC13164175; doi:10.3390/healthcare14091214)
Supplement: Supplementary file 1 [file healthcare-14-01214-s001.zip › File S2_MPS-2_Second round.pdf]

Dear Participants,

First, we would like to thank you for your prompt response and generous commentary contributions. The outcome of this first round/first section survey confirms strong consensus on key aspects from academic perspective as well as provides valuable insights on the variances in clinical practice highlighting potential areas for debate-based discussions and future exploration to bridge gaps between academic insights and clinical practice.

In this second round we kindly ask you to reconsider your answers on the academic statement items which couldn't reach the consensus threshold 80% but came close. Please see the statement items as below with their first-round results. Please do reconsider your answers if you didn't answer previously in favour of the outcome trend, but you are also welcome to keep your previously opinion as it is or change as you see fit. If you are not still convinced to change your opinion based on the first-round outcome, please kindly provide your reasoning for not doing so in the open-ended answer option which will give us valuable content to discuss within the study manuscript.

Section 1

Revisiting close to consensus statements.

1. **ERT should be started to patients who have severe form of the disease with mild neurological symptoms.** (First round statement and result: **ERT should be started to patients who have severe form of the disease with neurological symptoms** 20% strongly agree + 40% agree = **60%, close to consensus agreement**.)

- Strongly agree
- Agree
- Neither agree nor disagree
- Disagree
- Strongly disagree
- I also have an additional comment:

2. **ERT should be started to patients who have severe form of the disease with severe neurological symptoms.** (First round statement and result: **ERT should be started to patients who have severe form of the disease with neurological symptoms** First round result: 20% strongly agree + 40% agree = **60%, close to consensus agreement**.)

3. **The initiation of ERT in MPS II should be based on certain criteria <u>such as mobility, respiratory functions and cognitive impairment.</u> (**First round statement and result: **The initiation of ERT in MPS II should be based on criteria including mobility, respiratory functions and/or cognitive impairment.** 60% agree = **60%, close to consensus agreement**.)

4. **ERT should be started regardless of any criteria at any age.** (First round result: 20% strongly agree + 40% agree = **60%, close to consensus agreement**.)

5. **ERT should be started in MPS II patients over 24 months of age who has mobility without assistance and do not require respiratory support in the last six months, as part of the criteria for treatment eligibility. (**First round result: 20% strongly agree + 40% agree = **60%, close to consensus agreement**.)

6. **Haematopoietic Stem Cell Transplantation (HSCT) should be considered a viable treatment option for patients with attenuated MPS II. (**First round result: 10% strongly disagree + 40% disagree = **50% close to consensus disagreement**.)

7. **HSCT should be considered a viable treatment option in patients with severe MPS II. (**First round result: 10% strongly agree + 40% agree = 50% close to consensus agreement.)

8. **Substrate reduction therapy is a promising option for MPSII based on small-molecule inhibitors of GAG synthesis, which prevents substrate storage. (**First round result: 10% strongly agree + 60% agree = 70%, close to consensus agreement.)

9. **Gene therapy is an emerging treatment for MPSII and may replace ERT in the future.** (First round result: 70% agree = 70%, close to consensus agreement.)

Section 2

Seeking level of agreement on the most preferred answers by vast majority and on revised academic statements by putting actual clinical practices/capacities in consideration (aiming to find a common ground and not contradicting to the available evidence)

1. **The medical history evaluations and physical examinations should be performed every 6 months except case specific circumstances** (first round results: 80% selected every 6 months, 10% selected every 12 months, 10% every 3 months)

2. **Revised statement due to diverted opinions on Urinary GAG level monitoring:**

   It is a good clinical practice to monitor urinary GAG levelsat baseline and repeat every 6 months for the first year of ERT to track initial response and extend to 12 months once the patient's clinical and biochemical markers have stabilized, if there is aqequote clinical and test access capacity (first round results: 50% selected every 6 months, 30% selected every 12 months, 10% every 24 months, 10% no need for routine follow-up and can be done if there is suspicion for treatment ineffectiveness)

3. **Revised statement due to diverted opinions on liver and spleen size monitoring by ultrasound:**

   It is a good clinical practice to measure liver and spleen size by ultrasound at baseline and repeat every 12 months if there is aqequote clinical/imaging access capacity (except case specific circumstances where a more often monitoring is required) (first round results: 30% selected every 6 months, 60% selected every 12 months, 10% no need for routine follow-up if hepatosplenomegaly is not evident in physical examination)

4. **Revised statement due to diverted opinions on 6-Minute Walk Test monitoring:**

   It is a good clinical practice to perform the 6MWT at baseline and repeat every 12 months. (first round results: 40% selected every 6 months, 60% selected every 12 months,)

5. **Revised statement due to diverted opinions on monitoring with spirometry.**

   It is a good clinical practice to perform spirometry at baseline and repeat every 12 months if there is aqequote clinical/test access capacity. (except case specific circumstances where a more often monitoring is required). (first round results: 40% selected every 6 months, 60% selected every 12 months,)

6. **Revised statement due to diverted opinions on cognitive assessments:**

It is a good clinical practice to perform cognitive assessments in patients with MPS-2 every 6-12 months months until 3 years of age depending on phenotype severity and any emerging concerns. **(**first round results: 20% selected every 6 months, 60% selected every 12 months, 20% selected every 24 months)

7. **Related additional statement for above topic:**
   It is a good clinical practice to perform cognitive assessments in patients with MPS-2 every 12 months between 3-5 years of age if stable, more often if there are signs of rapid changes or concerns about regression.

8. **It is a good clinical practice to perform cognitive assessments in patients with MPS-2 every 12–18 months between 5-12 of age, focusing on academic performance, attention, adaptive functioning, and social development.**

9. **It is a good clinical practice to perform cognitive assessments in patients with MPS-2 every 2 years as off from 13 years of age individualized to the patient's clinical course and school/vocational needs.**

10. **Revised statement due to diverted opinions on Echocardiograms and ECGs assessments:**

    It is a good clinical practice to perform echocardiograms and ECGs at baseline and repeat every 12 months if there is aqequote clinical/test access capacity. (except case specific circumstances where a more often monitoring is required) **(**first round results: 30% selected every 6 months, 70% selected every 12 months,)

11. **Joint range of motion should be assessed at baseline and repeat every 12 months if there is aqequote clinical capacity. (except case specific circumstances where a more often monitoring is required.) (**first round results: 20% selected every 6 months, 80% selected every 12 months,)

12. **Revised statement due to diverted opinions on MRI of the spine and brain:**

    It is a good clinical practice to perform MRI of the spine (particularly cervical) **and the brain at the baseline and repeat every 24 months for patients with MPS-2 if there is aqequote imaging capacity. (except case specific circumstances where a more often monitoring is required.) (**first round results: 50% selected every 12 months, 40% selected every 24 months, 10% once at diagnosis is enough for detecting surgical problems)

13. **Revised statement due to diverted opinions on otolaryngologic follow-ups:**

    It is a good clinical practice to perform otolaryngologic follow-up at baseline and repeat every 12 months if there is aqequote clinical capacity. (except case specific circumstances where a more often monitoring is required) (first round results: 20% selected every 6 months, 70% selected every 12 months, 10% selected every 24 months)

14. **Revised statement due to diverted opinions on** ophthalmological examination with fundus assessments:

    It is a good clinical practice to perform ophthalmological examination with fundus assessments at baseline and repeat every 12 months if there is aqequote clinical capacity.(except case specific circumstances.) (first round results: 10% selected every 6 months, 60% selected every 12 months, 30% selected every 24 months)

15. **Revised statement due to diverted opinions on regular IgG monitoring:**
    It is a good clinical practice to perform IgG monitoring at baseline and repeat annually or more often if clinical response is inadequate or due to allergic reactions if there is aqequote clinical/test access capacity.. (first round results: 20% selected every 6 months, 30% selected every 12 months, 10% selected every 24 months, and 40% thinks it doesn't have to be measured regularly and can be measured if clinical response is inadequate or due to allergic reactions)

16. **Revised statement due to diverted opinions on regular polysomnography.**
    It is a good clinical practice to perform polysomnohgraphy at baseline and repeat annually in case of sleep apnoea or nocturnal snoring. (first round results: 10% selected every 6 months, 40% selected every 12 months, 40% selected every 24 months, and 10% thinks it doesn't have to be measured regularly and can be measured if sleep apnoea or nocturnal snoring is present or suspected)

17. **Quality of life questionnaires should be administered annually to evaluate the impact of ERT on patients' overall well-being.** (first round results: 10% selected every 6 months, 80% selected every 12 months, 10% selected every 24 months)

Section 3

Revisiting close to consensus statements.

1. **ERT should be discontinued or suspended when there is a severe infusion-associated reaction that cannot be managed with recommended premedication and desensitization. (First round results:** 40% strongly agree + 30% agree = **70%, close to consensus agreement**.)
2. **ERT should be discontinued or suspended when there are secondary life-threatening comorbidities (review on a case-by-case basis).** (First round result: 40% strongly agree + 20% agree = **60%, close to consensus agreement**.)
3. **ERT should be discontinued or suspended when there is progressive disease that does not improve or stabilise with ERT (First round statement and result: ERT should be discontinued or suspended when there is severe or advanced disease that does not improve with ERT.** 50% agree + 10% strongly agree = **Close to agreement consensus with split views** of disagreement)

4. **Revised statement:**
Lack of ongoing improvement or stabilization in the 6MWT should not be a sole condition to discontinue ERT. (First round statement and result: **There should be an ongoing improvement or stabilization in the 6MWT to continue ERT** : 60% disagree + 10% strongly disagree = 70%, **close to consensus disagreement.)**

5. **ERT should be discontinued if neurological decline progresses to a severe degree.** (20% strongly agree + 40% agree = **60%, close to consensus agreement**.)

6. **Revised statement based on additional comments by participants from first round:**
Echocardiographic functions (ejection fraction, fractional shortening, and myocardium thickness) should not be a criteria for treatment discontinuation. (First round statement and result: **Echocardiographic functions (ejection fraction, fractional shortening, and myocardium thickness) should be criteria for treatment discontinuation.** 40% disagree + 30% strongly disagree = **70%, close to consensus disagreement**.)

7. **Revised statement based on additional comments by participants from first round:**
While there are not enough evidence to directly recommend if ERT must be **discontinued or suspended during pregnancy or lactation in MPS II, if the clinical benefit is significant, continuing therapy—with appropriate monitoring—can be reasonable**. Ultimately, the decision is individualized, balancing the risks and benefits in consultation with the specialist team. (First round statement and result: **ERT should be discontinued or suspended when there is pregnancy or breastfeeding.** 30% agree where 40% disagree, diverted opinion**)**

8. **Revised statement based on additional comments by participants from first round:**

   Deciding whether to continue or suspend ERT in a patient with an unrelated, terminal illness depends on the overall goals of care, quality of life considerations, and the projected benefits of therapy. Ultimately, the decision is individualized, balancing the risks and benefits in consultation with the specialist team. (First round statement and result: **ERT should be discontinued or suspended when there is incurable disease unrelated to Hunter syndrome** (e.g., terminal cancer), 40% agree where 40% disagree, diverted..)

Seeking level of agreement on the most dominant opinions via revised academic statements (by also putting actual clinical practices in consideration) (aiming to find a common ground and not contradicting to the available evidence)

9. Inadequate response (inc any level of decline) in 6MWT follow up after any length of ERT **should not solely be considered as treatment failure.**

10. Inadequate response (inc any level of decline) in respiratory function tests (FVC) follow up after any length of ERT **should not solely be considered as a treatment failure nor indicate treatment discontinuation.**
11. Inadequate response (inc any level of increase) on liver or spleen size or volume up after any length of ERT **should not solely be considered as treatment failure nor indicate treatment discontinuation.**
12. Individualised and overall assessment, together with the physician's, patient's and the family's opinion should be the determinants of ERT discontinuation.
13. ERT continuation is important due to the absence of any other specific treatment. The criteria should be regulated in a positive way to improve the patient's quality of life.

Section 4

Revisiting close to consensus statements.

1. **New statement based on additional comments by participants from first round:** While premedication and desentisation to prevent hypersensitivity reactions in ERT is not mandatory for all cases, it is good clinical practice to individualize protocols, tailor to the patient's infusion-related reaction history, comorbidities, and risk factors.

2. **Antihistamines and corticosteroids are essential components of the premedication regimen before ERT in MPS II patients to prevent hypersensitivity reactions. (**First round result: 40% agree + 20% strongly agree = **60%, close to consensus agreement**.)

3. **Revised statement based on additional comments by participants from first round:**

   Home infusion of ERT for MPS II can be considered for children older than 2 years of age, provided they have had no significant infusion reactions in the preceding 6 months and there is a trained healthcare professional available to manage potential emergencies. (**First round statement and result: Home infusion of ERT should be considered for children older than older than 2 years of age.** 40% agree + 20% strongly agree = **60%, close to consensus agreement**.)

4. **Revised statement based on additional comments by participants from first round:**

   Home infusion of ERT for MPS II can be considered for children older than 5 years of age, provided they have had no significant infusion reactions in the preceding 6 months and there is a trained healthcare professional available to manage potential emergencies . **(First round statement and result: Home infusion of ERT should be considered for children older than 5 years of age.** 60% agree + 10% strongly agree = **70%, close to consensus agreement**.

5. **Revised statement based on additional comments by participants from first round:**

   The national medical association should lead the initiative in overseeing and maintaining the MPS II registry and monitor the disease together w**ith the health authority** to ensure data accuracy, compliance with national standards w**ith ethical measures and permissions** taken. (**First round statement and result: The national medical association should lead the initiative in overseeing and maintaining the MPS II registry to ensure data accuracy and compliance with national standards.** 40% agree + 30% strongly agree = **70%, close to consensus agreement**.)
