# Supplementary material for: Harmonizing Perspectives on MPS II Care in Türkiye: A Delphi Study Towards Treatment Management Consensus
Source: Healthcare (Basel). 2026 Apr 30;14(9):1214. doi: 10.3390/healthcare14091214 (PMC13164175; doi:10.3390/healthcare14091214)
Supplement: Supplementary file 1 [file healthcare-14-01214-s001.zip › File S3_MPS-2_Delphi first Round Report.pdf]

# Report on MPS II Treatment Management in Turkey - First Round Delphi Survey

Survey results are shared in 4 different pdf documents attached to the same email. **Consensus Agreement**, **Consensus Disagreement**, and **Close to Consensus** based on the first-round survey data and the following definitions:

1. **Consensus Agreement**: Agree + Strongly Agree ≥ 80%.
2. **Consensus Disagreement**: Disagree + Strongly Disagree ≥ 80%.
3. **Close to Consensus**: Agree + Strongly Agree is below 80% but equal or above 50% or Disagree + Strongly Disagree is below 80% but equal or above 50%.

In this word document we intentionally included only: **close to consensus items** as well as **debatable statements** due to diverted opinions and **important insights where there is significant variation** from the provided academic opinions on given statements, **additional important notes** mutually provided by the participants which will be used to finalise second round questions.

---

**Section 1: Considerations on treatment initiation: when and whom to treat? (inc; understanding disease severity and progression predictions)**

---

## Summary

The outcome of this first round/first section survey confirms strong consensus on key aspects like early ERT initiation, palliative care integration, and multidisciplinary care. Disagreements highlight differing opinions on the necessity of ERT for mild or severe symptoms and the perceived role of palliative care. Variances in clinical practice highlight cautious applications of ERT for severe neurological symptoms and cognitive impairments, while emerging therapies like gene therapy and substrate reduction therapy represent potential areas for future exploration to bridge gaps between academic insights and clinical practice.

**Close to Consensus Items**

1. **Q7. ERT should be started to patients who have severe form of the disease with neurological symptoms.**
   - 20% strongly agree + 40% agree = **60%, close to consensus agreement**.
2. **Q12. ERT should be started regardless of any criteria at any age.**
   - 20% strongly agree + 40% agree = **60%, close to consensus agreement**.
3. **Q13. The initiation of ERT in MPS II should be based on criteria including mobility, respiratory functions and/or cognitive impairment.**
   - 60% agree = **60%, close to consensus agreement**.
4. **Q14. ERT should be started in MPS II patients over 24 months of age who has mobility without assistance and do not require respiratory support in the last six months, as part of the criteria for treatment eligibility.**
   - 20% strongly agree + 40% agree = **60%, close to consensus agreement**.

5. **Q18. Haematopoietic Stem Cell Transplantation (HSCT) should be considered a viable treatment option for patients with attenuated MPS II.**
    - 10% strongly disagree + 40% disagree = **50% close to consensus disagreement**.
6. **Q19. HSCT should be considered a viable treatment option in patients with severe MPS II.**
    - 10% strongly agree + 40% agree = **50% close to consensus agreement**.
7. **Q22. Substrate reduction therapy is a promising option for MPSII based on small-molecule inhibitors of GAG synthesis, which prevents substrate storage.**
    - 10% strongly agree + 60% agree = **70%, close to consensus agreement**.
8. **Q23. Gene therapy is an emerging treatment for MPSII and may replace ERT in the future.**
    - 70% agree = **70%, close to consensus agreement**.

---

**Debatable statements due to diverted opinions.**

1. **Q8. ERT should be started to all patients regardless of the severity and CNS involvement. (40% to ,40 %)**

---

**Insights from Clinical Practice Questions**

This section evaluates clinical practices and compares them with academic consensus outcomes.

---

**Practice Differences (Divergences from Consensus)**

1. **Q32. ERT for severe neurological symptoms should always be administered.**
    - Only 20% always administer ERT to patients with severe neurological symptoms in practice, despite broader academic support.
2. **Q36. ERT should be administered irrespective of cognitive impairment.**
    - Only 20% of respondents administer ERT irrespective of cognitive impairment in practice, diverging from academic favourability.
3. **Q43. Gene therapy should be considered as a potential future option for treatment.**
    - 60% would often consider using gene therapy if available, reflecting interest in emerging academic insights, although it is not yet implemented in practice.
4. **Q42. Substrate reduction therapy should be considered as a therapeutic option.**
    - No respondents reported using substrate reduction therapy, despite its academic promise.

Important additional comments for publication manuscript:
**ERT have not significantly improved the CNS manifestations of patients thus far. Patients with damaged nervous systems benefit little from HSCT. New treatments that can penetrate the BBB, such as gene therapy and SRT are under active research, which**

**provides hope for severe form of the disease in the future. Further research should focus on investigating the efficacy of combination therapies.**

**HSCT may improve neurological symptoms. Compared with ERT, HSCT produces enzymes more continuously, does not require regular supplementation, and can cross the BBB to produce enzymes in brain tissue, improving neurological symptoms and lowering the relative medical cost. But in my practice, I prefer ERT in patients with MPS II. There are certain risks associated with HSCT including postoperative infection, transplant rejection and graft-versus-host disease. Before treatment I tell the risks and benefits of ERT and HSCT for severe form of the disease.**

---

**Section 2: Considerations on follow up and monitoring.**

---

## Summary

The second section of the survey results highlights the following:

- **Close to Consensus Items**: Practices such as measuring urinary GAG levels, liver and spleen ultrasounds, joint range of motion, and polysomnography assessments exhibit variability. Close to consensus indicates ongoing divergence in practices.
- **Alignment with Consensus**: Respondents frequently align with their academic beliefs on semi-annual evaluations for follow-ups, spirometry, and 6-Minute Walk Tests. Otolaryngologic and ophthalmological follow-ups also align closely with academic expectations.
- **Practice Gaps**: Discrepancies in the frequency of urinary GAG measurements, liver and spleen size monitoring, polysomnography and quality of life assessments highlight areas where clinical practice diverges from academic recommendations. Addressing these gaps may improve the standardization of MPS II treatment and outcomes.

**Close to Consensus Items**

1. **Q50. How often should urinary GAG levels be measured?**
   - 50% every 6 months + 30% every 12 months
2. **Q51. How often should liver and spleen size be measured by ultrasound?**
   - 30% every 6 months + 60% every 12 months
3. **Q52. How often should the 6-Minute Walk Test (6MWT) be performed?**
   - 40% every 6 months + 60% every 12 months **diverging on annual versus biennial assessments**.
4. **Q54. How often should cognitive assessments be conducted?**
   - 20% every 6 months + 60% every 12 months
5. **Q55. How often should echocardiograms and ECGs be performed?**
   - 30% every 6 months + 70% every 12 months

6. **Q57. How often should an MRI of the CNS be performed for patients with MPS II?**
    - 50% every 12 months + 40% every 24 months **diverging on annual versus biennial assessments**.
7. **Q58. How often should an MRI of the spine be performed for patients with MPS II?**
    - 40% every 12 months + 50% every 24 months **diverging on annual versus biennial assessments**.
8. **Q59. How often should an otolaryngologic follow-up be recommended?**
    - 20% every 6 months + 70% every 12 months
9. **Q60. How often should an ophthalmological examination with fundus assessments be conducted?**
    - 60% every 12 months + 30% every 24 months **diverging on annual versus biennial assessments**.

---

**Debatable statements due to diverted opinions.**

1. **Q61. How often should regular monitoring of Immunoglobulin G (IgG)antibodies to ERT be done?**

- Very variable answers, where 40% thinks it doesn't have to be measured regularly and can be measured if clinical response is inadequate or due to allergic reactions.

2. **Q62. How often should polysomnography be performed to assess sleep quality and respiratory function in patients receiving ERT?**

    - 40% every 12 months + 40% every 24 months

---

**Insights from Clinical Practice Questions**

This section evaluates clinical practices and identifies patterns that align with or diverge from consensus academic views.

---

**Practice Differences (Divergences from Consensus)**

1. **Q67. In my clinic, urinary GAG levels are measured every 6 months.**
    - Only 40% of respondents follow this practice, which is less frequent than academic recommendations.
2. **Q68. In my clinic, liver and spleen size are measured by ultrasound every 12 months.**
    - 50% of respondents conduct annual ultrasounds, which is less frequent than academic expectations for semi-annual monitoring.
3. **Q69. In my clinic, liver and spleen size are measured by MRI**

**New statement item:** If USG measurement is not sufficient, volumetric measurement is required, or clinically indicated then MRI should be utilised but not as a regular assessment.

4. **Q70. In my clinic, the 6-Minute Walk Test (6MWT) is performed:**
   - 70% of respondents conduct the test every 6 months which is more frequent than academic expectations for annual monitoring.
5. **Q74. In my clinic, joint range of motion assessments are conducted every 12 months.**
   - Only 50% of respondents perform annual assessments, diverging from academic consensus for annual evaluations.
6. **Q78. In my clinic, ophthalmological examinations with fundus assessments are conducted.**
   - 80% of respondents perform annual assessments despite the unachieved consensus with 60% agreement on annual examination?
7. **Q82. In my clinic, quality of life questionnaires are administered every 18–24 months to evaluate the impact of ERT on patients' overall well-being.**
   - Only 50% of respondents conduct annual assessments, despite the consensus achieved.

---

**Section 3: Considerations on treatment discontinuation: defining stopping points.**

---

## Summary

The results from Section 3 reveal the following key findings:

1. **Close to Consensus Items**:
   - Topics such as ERT discontinuation for severe infusion-associated reactions, life-threatening comorbidities, and neurological decline showed variability among participants.
2. **Alignments with Academic Consensus**:
   - Clear alignment is seen in areas like using overall health assessments for continuing ERT and recognizing that ERT is well-tolerated with mild to moderate side effects.
3. **Divergences from Consensus**:
   - Significant variations were observed in clinical decisions on ERT discontinuation due to life-threatening comorbidities, advanced disease progression, and neurological decline.

Bridging these divergences between clinical practices and academic recommendations will require further dialogue and agreement to standardize MPS II management strategies.

**Close to Consensus Items**

1. **Q86. ERT should be discontinued or suspended when there is a severe infusion-associated reaction that cannot be managed with recommended premedication.**
    - 40% strongly agree + 30% agree = **70%, close to consensus agreement**.
2. **Q87. ERT should be discontinued or suspended when there are life-threatening comorbidities (review on a case-by-case basis).**
    - 40% strongly agree + 20% agree = **60%, close to consensus agreement**.
3. **Q90. ERT should be discontinued or suspended when there is severe or advanced disease that does not improve with ERT.**
    - 50% agree + 10% strongly agree = **Close to consensus with split views**.
4. **Q93. There should be an ongoing improvement or stabilization in the 6MWT to continue ERT.**
    - 60% disagree + 10% strongly disagree = **70%, close to consensus disagreement**.
5. **Q96. ERT should be discontinued if neurological decline progresses to a severe degree.**
    - 20% strongly agree + 40% agree = **60%, close to consensus agreement**.
6. **Q97. Echocardiographic functions (ejection fraction, fractional shortening, and myocardium thickness) should be criteria for treatment discontinuation.**
    - 40% disagree + 30% strongly disagree = **70%, close to consensus disagreement**.

---

**Debatable statements due to diverted opinions.**

**Q88. ERT should be discontinued or suspended when there is pregnancy or breastfeeding.**

- 30% agree where 40% disagree, diverted..

**Q89. ERT should be discontinued or suspended when there is incurable disease unrelated to Hunter syndrome (e.g., terminal cancer)**

- 40% agree where 40% disagree, diverted..

**Q92.What is the optimal length of treatment with ERT before an inadequate response in the 6MWT may be considered as treatment failure? 50% responded in comments as:** 6MWT should not be used as the sole indicator of treatment failure. Inadequate response in the 6MWT should not be considered as treatment failure.

**Q94. What decline in the annual 6MWT indicates that ERT should be discontinued?**

a severe CNS involvement should be the only indication, Inadequate 6MWT alone should not be used to evaluate treatment unresponsiveness. 6MWT cannot be a criterion alone; it should be evaluated together with other clinical recovery conditions.

**Q98.What is the optimal length of treatment with ERT before an inadequate response in echocardiographic functions (ejection fraction, fractional shortening, and myocardium thickness) may be considered as treatment failure?**

- 50% 24 months, the rest commented: Poor response in echocardiogram alone does not equal treatment failure. Decision for treatment failure should not be based on only echocardiographic functions. Progression of cardiac involvement varies from patient to patient. Worsening of cardiac involvement may occur under ERT treatment.

**Q101. What is the optimal length of treatment with ERT before an inadequate response in respiratory function tests (FVC) may be considered as treatment failure?**

- 40% 24 months, the rest commented: Non-response to treatment cannot be attributed solely to the lack of expected improvement in respiratory function tests. There are also non-pulmonary factors that affect respiratory function tests. It should be evaluated together with clinical findings.

**Q102. What decline in FVC indicates that ERT should be discontinued?**

40% responded more than 30%, rest commented: No such cut-off should exist.

**Q104.What is the optimal length of treatment with ERT before an inadequate response in liver and spleen size and volume may be considered as treatment failure?**

**30% said 12 months, 40% said 24 months, where others commented:** For treatment failure the criteria could not be only volume of liver and spleen. Liver and spleen size does not have significant contribute onto disease morbidity in MPS-II. It should not be considered as a treatment discontinuation criterion.

**Q105. What increase in liver or spleen size or volume indicates that ERTshould be discontinued?**

40% said more than 30%, 60% commented: Decision for ERT discontinuation should not be based on only organ volumes.

**Important additional comments:**

- In our clinical practice, we did not experience any serious adverse events that required discontinuation of ERT. ERT continuation is important due to the absence of any other specific treatment. The criteria should be regulated in a positive way to improve the patient's quality of life.
- It is possible that some patients have significant impairment in a certain organ or system involvement, which improves and drastically improves the patient's quality of life, while changes (positive or negative) in other systems may be negligible - this should not prevent patients from continuing. Individualised and overall assessment, together with the physician's, patient's and the family's opinion should be the determinants of discontinuation.

**Insights from Clinical Practice Questions (Q109–Q125)**

This section evaluates clinical practices and compares them with academic consensus outcomes.

---

**Key Practice Patterns Aligning with Consensus (which will be important to validate in the second round)**

1. **Q109. In my clinic, I discontinue or suspend ERT when there is a severe infusion-associated reaction that cannot be managed with recommended premedication.**
   - 40% always + 10% often = **50%, close to alignment with academic opinion** but 40% rarely or never?
2. **Q111. In my clinic, I discontinue or suspend ERT when there is pregnancy or breastfeeding.**

   70 %rarely or never discontinue in real life close to academic opinion

3. **Q112. In my clinic, I discontinue or suspend ERT when there is incurable disease unrelated to Hunter syndrome (e.g., terminal cancer).**
   - 60% rarely or never = **Aligned with case-by-case approach recommendation outcome (Q89)**
4. **Q122. My decision for continuing ERT is based on an overall assessment including growth, organ involvement, quality of life.**
   - 100% always = **Full alignment with academic consensus**.
5. **Q124. I observe that ERT is well tolerated, and most side effects are mild to moderate.**
   - 70% always + 30% often = **Fully aligned with academic observations**.

---

**Practice Differences (Divergences from Consensus)**

---

Vast majority of the participants do not discontinue nor suspend ERT treatment in their clinical practice based on solo assessment criteria but on overall clinical assessment/outcome. However there is a significant diverted opinion on FVC, liver and spleen size/assessment frequence, neurological decline as half of the participants selected some criteria and timeline where the rest didn't**. These insights are also indicating and divergence from the SUT stopping criteria (more than 30% decline from the base evaluations: echo, 6MWT, spleen size etc) , which may not be worded in the article due to sensitivity.**

---

**Section 4: Considerations on improving clinical management approach: improving clinical governance, adherence and persistence.**

## Summary

Topics like premedication protocols, post-infusion monitoring, patient transitions, and establishing centralized registries require further discussion to achieve broader consensus among experts. These results underline the need for continued dialogue and alignment to ensure best practices for MPS II treatment management.

**Close to Consensus Items**

- **Q127. Antihistamines and corticosteroids are essential components of the premedication regimen before ERT in MPS II patients to prevent hypersensitivity reactions.**

  - 40% agree + 20% strongly agree = **60%, close to consensus agreement**.

Important note: Patients without a history of infusion-related reactions do not always require premedication. Premedication does not prevent and may even mask early symptoms of an anaphylaxis (eg. urticaria) corticosteroids are essential components of the premedication regimen before ERT in some MPS II patients according to their history We do not use steroids routinely to every patient

**Q139. Home infusion of ERT should be considered for children older than 2years of age**

- 40% agree + 20% strongly agree = **60%, close to consensus agreement**.

**Q140. Home infusion of ERT should be considered for children older than 5years of age.**

- 60% agree + 10% strongly agree = **70%, close to consensus agreement**.

**Q141. Home infusion of ERT should not be an option for children.**

- 50% disagree + 20% strongly disagree = **70%, close to consensus disagreement**.

Important notes: -Home infusions can be considered if patients have had no reactions > 6 months, provided that there is a health care team confident with establishing an IV line, administering the infusion and treating any life-threatening hypersensitivity reactions (including intubation or cricothyroidotomy, which can be very difficult in patients with MPS-II). Home infusions should be implemented, but the healthcare system should provide adequate staff training to take the necessary precautions and follow-up. There are studies showing the safety of home treatment. There are countries that apply this method. The decision is made by evaluating the patient's clinical condition and history of adverse events during treatment.

**Q149. The national medical association should lead the initiative in overseeing and maintaining the MPS II registry to ensure data accuracy and compliance with national standards.**

- 40% agree + 30% strongly agree = **70%, close to consensus agreement**.

Important note: Registry systems containing data should be under the control of a commission consisting of metabolic physicians who monitor the disease together with the national health authority, with ethical measures and permissions taken.

---

End of Document
